# A conserved acidic residue drives thyroxine synthesis within thyroglobulin and other protein precursors

**DOI:** 10.1016/j.jbc.2024.108026

**Published:** 2024-11-26

**Authors:** Camilla Stejskalova, Federica Arrigoni, Riccardo Albanesi, Luca Bertini, Luca Mollica, Francesca Coscia

**Affiliations:** 1 Human Technopole, Structural Biology Centre, Milano, Italy; 2 Department of Biotechnology and Biosciences, University of Milano-Bicocca, Milano, Italy; 3 Department of Medical Biotechnology and Translational Medicine, Università degli Studi di Milano, Milano, Italy

**Keywords:** density functional theory, molecular dynamics, iodination, thyroglobulin, thyroid hormones, thyroxine, protein engineering

## Abstract

Thyroxine, the main hormone product of the thyroid, is produced at multiple sites within its protein precursor thyroglobulin. Each site consists of two tyrosine residues which undergo iodination and coupling, resulting in the synthesis of thyroxine at the acceptor tyrosine, where the hormone synthesis is later completed by proteolysis. Within the structurally resolved sites, the role of an essential conserved acidic residue preceding the acceptor remains elusive. To elucidate the mechanism of thyroxine synthesis we engineered a single-site minimal protein precursor. First, by its *in vitro* iodination and site-directed mutagenesis we show that the presence of the acidic residue, preferably glutamate, favors thyroxine synthesis. Secondly, within the designed precursor, we computationally modeled the reaction of iodination and iodotyrosine coupling giving rise to thyroxine. Our results reveal that hormone formation is triggered by iodotyrosine deprotonation, facilitated by proximity to a carboxylic group, closer in the case of glutamate, in line with our experimental findings and sequence conservation. Hereafter, we surmise that in the natural precursor thyroglobulin, two evolutionary late and slower hormonogenic sites coexist with an early evolutionary and faster one. Indeed, the latter is overlapping with a proteolytic site, thereby allowing prompt thyroxine release from thyroglobulin.

Thyroid hormones (TH) are essential regulatory iodo-compounds at the center of vertebrate metabolism, and their imbalance is related to several human diseases (1). The mechanistic understanding of TH synthesis and regulation is still poor, partly due to the unusual biochemistry involving iodine and the limitations of the available methods for its analysis. Thyroxine (3,5,3′,5′ tetraiodo-L-thyronine, or T4) is the main hormone product of the thyroid gland, and it is secreted in the bloodstream to reach other target tissues (*e.g.*, brain, liver, kidney), where it can undergo deiodination to generate the more active form T3 (3,3′,5-triiodo-L-thyronine) and other products. Notably, T4 is also used to treat thyroid hormone deficiency (2, 3, 4). Within the thyroid, T4 synthesis occurs *via* iodination of its complex protein precursor thyroglobulin (TG), conserved in all vertebrates (5, 6, 7). In TG, a T4 hormonogenic site is made by two tyrosine residues, which are enzymatically iodinated to two di-iodo-tyrosines (3,5 diiodotyrosine; DIT). Then, the hydroxyl group -OH of one DIT (*via* its Oζ) couples to the Cβ of the second DIT, resulting in the synthesis of T4 (acceptor DIT position) and a dehydroalanine (donor DIT position) (Fig. 1). T4, embedded in the polypeptide chain of the protein precursor, is later released by proteolysis mainly by cathepsin enzymes, located in lysosomes and partly in the extracellular lumen of the thyroid follicles (8, 9). The recently solved mammalian TG three-dimensional structure clarified which are the tyrosine pairs (acceptors and donors) forming T4 in the four hormonogenic sites A-D (10, 11, 12, 13). Intriguingly, the studies indicated that these TG sites are structurally diverse, *i.e.*, the tyrosine pairs are not in a conserved pocket, but are only exposed, flexible, and proximal to allow T4 synthesis upon iodination. Indeed, hormone synthesis could be recapitulated by *in vitro* iodination of a maltose binding protein (MBP) scaffold simply bearing a flexible, proximal, and solvent-accessible tyrosine pair (10). Reflecting the average features of TG sites, the engineered site bears a structured donor tyrosine in an exposed alpha helix (Y341) and the acceptor (375) in a C-terminal flexible region. In addition, mutagenesis studies on human TG revealed that in the resolved sites A, B, and D, the consensus sequence Glu/Asp-Tyr at the acceptor position, absent at the donor, is essential for T4 synthesis (10, 14, 15). Currently, it is still unclear which tyrosine behaves as an acceptor within the hormonogenic pair, and this aspect is crucial since the final position of T4 is the substrate for its controlled release by proteolysis. Because the directionality and yields of the hormonogenesis reaction could not be inferred from the TG structures and the mechanism of T4 synthesis is still poorly characterized, it is difficult to make any biochemical or evolutionary hypotheses on this important aspect of TH biogenesis. In addition, the process is particularly arduous to investigate within the large, multimeric, and pluri-hormonogenic scaffold of TG.

**Figure 1:**
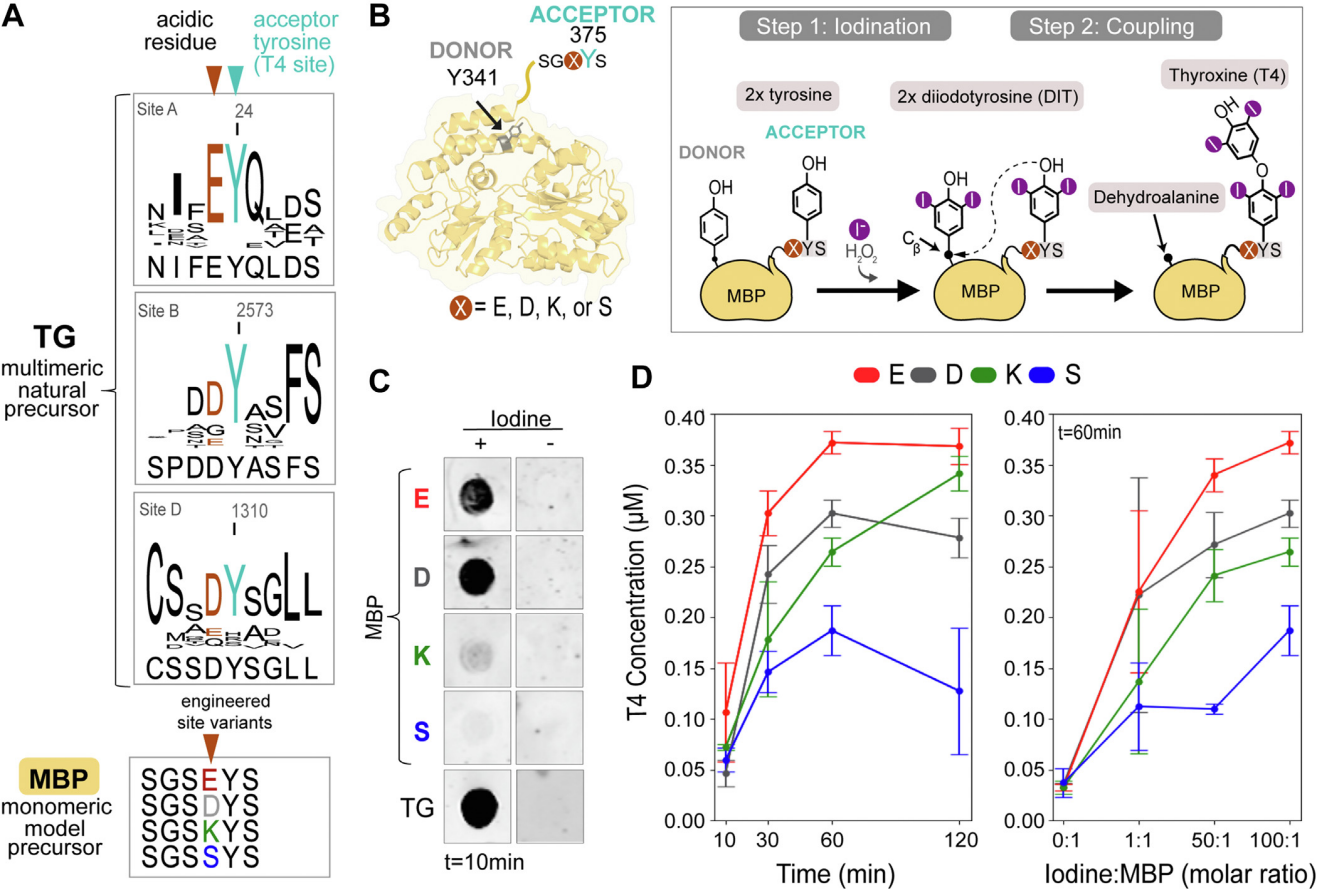
***In vitro* T4 synthesis on a model precursor.***A*, sequence conservation of hormonogenic sites (*A*), (*B*), and (*D*) across vertebrate TG. The consensus shows that at site *A* the acceptor tyrosine is always preceded by a glutamate, while in the evolutionary late sites (*B*) and (*D*) the consensus sequence favors an aspartate. The lower panel indicates the mutants of the MBP precursor that have been engineered to investigate the impact of the residue preceding the acceptor on T4 synthesis. *B*, schematics showing the MBP model precursor (*yellow*) and its engineered hormonogenic tyrosine pair (Y341 donor in gray and acceptor Y375 in teal), together with the general overview of T4 synthesis steps. *C* and *D*, dot blot, and T4-ELISA analysis of iodinated MBP variants indicate that E/D facilitate T4 synthesis over K/S at residue 374. Measurements were performed in three biological replicates.

In this work, we study the mechanism of T4 synthesis in an optimized monomeric model precursor derived from MBP, *via* a hybrid experimental and computational methodology. First, we experimentally quantified T4 yields varying the nature of the residue preceding the acceptor by site-directed mutagenesis, the reaction time, and iodine concentration. In parallel, we performed Molecular Dynamics (MD) simulations for the same model precursor variants to outline trajectories of the two reactive tyrosine, following how they approach in space and in the absence and presence of the iodine moiety. From this initial *in silico* estimate of the mechanisms of iodination, we identified the most probable tyrosine pair conformations and performed Density Functional Theory (DFT) calculations to characterize the mechanism of tyrosine coupling and its intermediates. Our computational analysis reveals the nature and pH limits of T4 synthesis and explains the role of the conserved primary sequence of TG as a trigger for directional hormone formation. Taken together, we can recapitulate *in silico* the same relative ranking in making T4 by the MBP variants as observed *via in vitro* iodination. Hence, the presented characterization of T4 synthesis provides a framework to further rationalize thyroid hormone synthesis and regulation from its natural protein precursor in various cellular and biochemical conditions (16, 17).

## Results

### *In vitro* T4 synthesis by iodination of a model monomeric precursor

To analyze the sequence conservation flanking the hormonogenic tyrosine pairs, we aligned the human TG (hTG) protein sequence with 14 additional vertebrate species of similar sequence length (Figs. 1*A* and S1). While there is no evident conservation regarding the donor tyrosine sites, the acceptor tyrosines are consistently preceded by an acidic residue within the structurally resolved sites A, B, and D. In the most conserved, reactive, and exposed site A the acceptor (Y24 in hTG) is always preceded by a glutamate residue. For both sites B (acceptor Y2573 in hTG) and D (acceptor Y1310 in hTG and in vertebrates appearing from amphibians), an aspartate residue is present (Fig. 1*A*). To dissect the impact of this conserved acidic residue on T4 synthesis, we took advantage of an existing model monomeric precursor derived from MBP (reference structure PDBID 1ANF) (10). First, we replicated the highly conserved TG site A on MBP by extending it at the C-terminus. This MBP extension featured a pseudo acceptor tyrosine, Y375, preceded by E374 (Fig. 1*A*). We utilized an existing solvent-exposed tyrosine at position 341 as a pseudo-donor (Fig. 1*B*). We then performed site-directed mutagenesis to mutate to phenylalanine un-specifically iodinated tyrosines and performed mass spectrometry analysis of the final iodinated construct. We verified that our new model precursor directionally forms T4 at Y375, while Y341 converts to dehydroalanine (Fig. S2), and that this is the unique hormonogenic site within the protein. Starting from this optimized MBP construct, we mutated the residue preceding the acceptor E374 to D (conserved at sites B and D of TG), to K (of opposite charge), and to S (small neutral residue). When subjecting the MBP variants to *in vitro* iodination for 10 min and in large iodide molar excess, qualitative dot blot clearly indicated that T4 synthesis is favored when residue 374 is acidic (Fig. 1*C*). Quantitative T4 detection by ELISA on iodinated samples showed that all variants can form the thyroid hormone, but with progressively increasing rates when the residue 374 is S, K, D, E respectively (Fig. 1*D*). Moreover, we observed that an approximative plateau for T4 production is reached at a 60 min iodination reaction time. The relative rating of the mutants is kept constant when iodide molar excess is increased, and this is in line with the sequence conservation in the natural precursor TG. Our experimental outcomes establish that the engineered MBP is a robust thyroid hormone model precursor, enabling further investigations of the effect of residue 374 on T4 yields.

### Coupling propensity is observed for both tyrosine and DIT pairs

**Figure 2:**
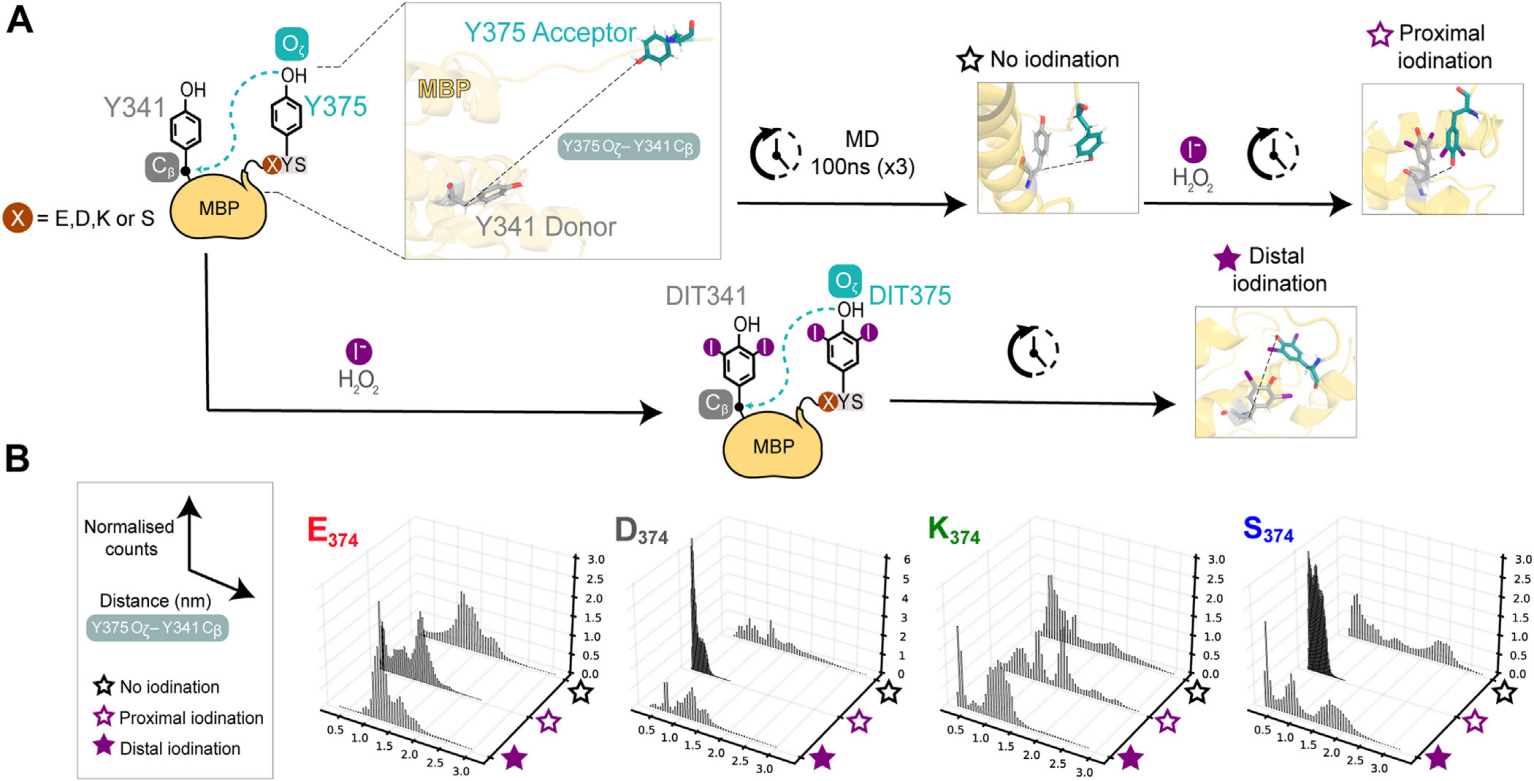
**Molecular Dynamics simulations of T4 synthesis within model precursor variants.***A*, Molecular Dynamics simulations of the model T4 precursor MBP, showing starting and representative frames of iodinated, distally iodinated (DIT from the start of the simulation) and proximally iodinated (iodinated at the most probable conformation) hormonogenic tyrosine pairs. *B*, probability distributions tracking the tyrosine-tyrosine distance O_ζ_-C_β_ representative of the coupling process, for every MBP variant (where the residue preceding the acceptor 374 is a E, D, K, S) and in the absence of presence of presence of the iodine moiety. The time-dependent graphs are reported in Figs. S5–S7.

To elucidate the impact of residue 374 on T4 yields, we sought to describe thyroid hormone synthesis *in silico* by Molecular Dynamics (MD) simulations. We statistically inspected the configurations of the C termini of the MBP constructs in terms of the Y375-Y341 tyrosine pair relative spatial orientations, following C_β_- C_β_ and O_ζ_-C_β_ interatomic distances respectively (see Fig. S4 for the nomenclature), in absence of iodine (tyrosine Y), including two iodine atoms at the Y-Y most stable proximal conformation (DIT, proxy iodination) or from the starting point of the dynamics and a maximized distance between iodinated tyrosines (DIT, distal iodination). Our analysis indicates that in all mutants, acceptor and donor tyrosine residues are statistically able to come closer in space when they are unbiasedly left free to explore the total conformational space of the system, *i.e.*, independently from the starting coordinates and the presence of the iodine moiety (Fig. 2). All the monitored distances present a broad distribution, indicating that the proximity between the tyrosine pair is compatible with the possibility of a reaction occurring in a statistically relevant manner. This supports the stochastic nature of the process and, consequently, the necessity of stabilizing any potential intermediate that could arise from this configuration. The presence of glutamate at position 374 (preceding the acceptor) seems to inhibit the stabilization of the Y-Y coupling, *i.e.*, allowing only a reduced number of configurations with C_β_- C_β_ and O_ζ_-C_β_ interatomic distances compatible with a spontaneous reactivity.

The sampling of structures with DIT in proximal configurations reveals that the presence of aspartate and serine stabilizes the system, strongly reducing the spread of distances. Conversely, when residue 374 is glutamate or lysine, it seems to destabilize the formation of the proximal pair, still displaying a spread of the monitored interatomic distances compatible with those sampled for simulations started from a distant Y-Y configuration. In all cases, a deeper and visual inspection of individual trajectories revealed the presence of stable conformations that are compatible with π−π stacking interactions between tyrosine sidechains, *i.e.*, at a distance between aromatic rings of ∼3.6 Å (18). An illustrative representation of the Y341-Y375 pair trajectory for the MBP E374 variant (MD simulation frames 1–450) is shown in Video S1.

### DIT coupling is a concerted and radical reaction

**Figure 3:**
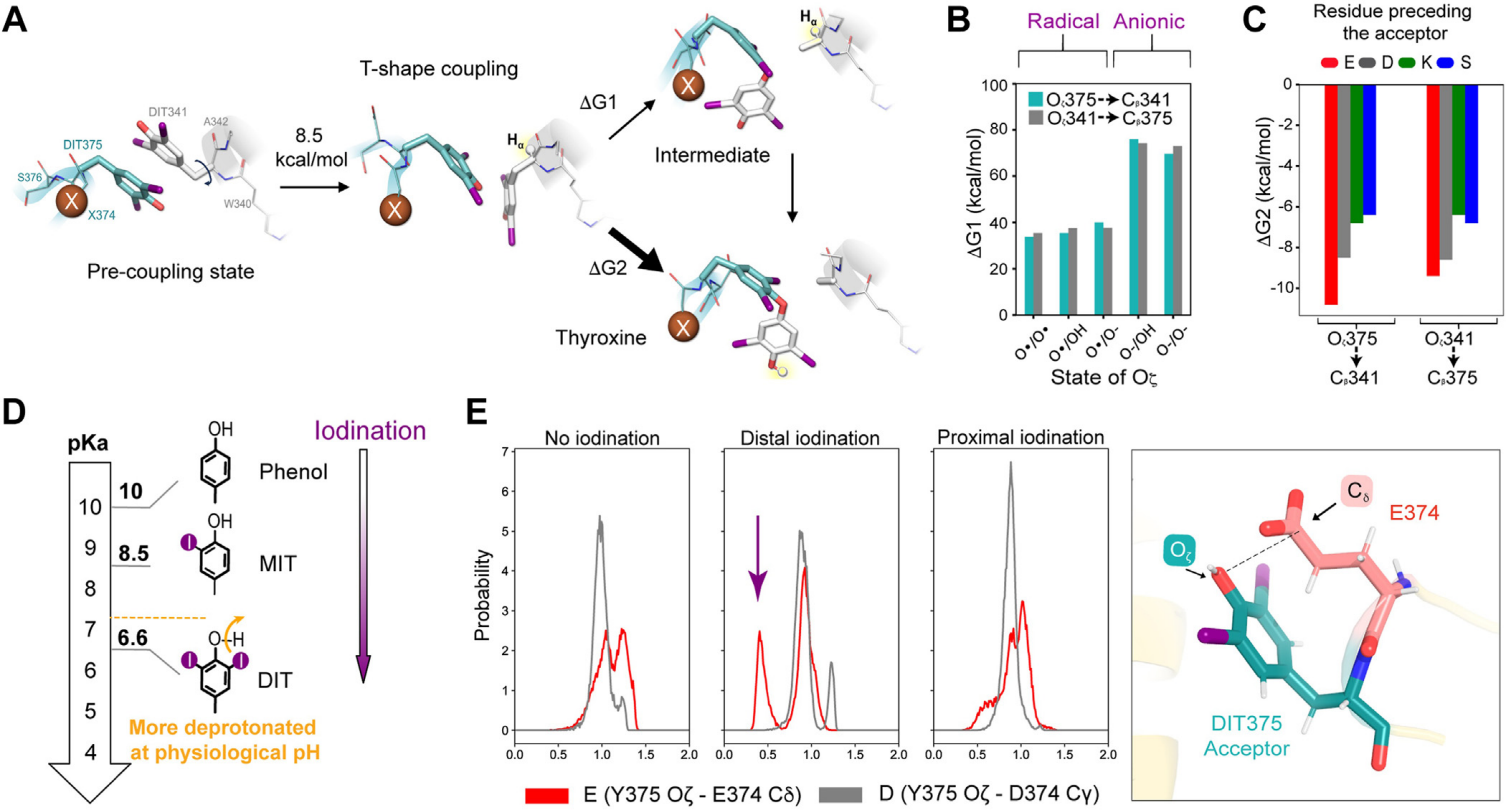
**Mechanism of T4 synthesis derived by *in silico* simulations of the MBP model precursor.***A*, calculated mechanisms for DIT coupling following a sequential (ΔG1) or concerted (ΔG2) route. Both mechanisms are triggered by a preliminary parallel to-perpendicular stacking rearrangement of the DITs couple (see Fig. S9 for details). *B*, ΔG1 values calculated for the MBP variant E374 considering a radical, anionic, and hybrid process coming from the donor (Y341) or as the real system from the acceptor (Y375). Radical processes are way more favored over anionic ones (see Figs. S8 for further details). *C*, ΔG2 values were calculated for all MBP mutants, showing that, in line with experimental data, the reaction is facilitated by the presence of a E, D, K, S preceding the acceptor in this order. *D*, estimated pKa for MIT and DIT, taking phenol as reference (see Fig. S12 for details). *E*, the probability distribution of distances (expressed in Angstrom) between the hydroxyl group oxygen atom of the acceptor tyrosine 375 (O_ζ_) and the carbon atom belonging to the carboxylic moiety of the sidechain of the acidic residue 374 (*i.e.*., C_δ_ of glutamate in red, C_γ_ of aspartate in green). The distributions have been computed for the three configurations and iodination conditions inspected in the present study and described in the Experimental Procedures section, *i.e.*, from left to right, distal non-iodinated tyrosines, distal iodinated tyrosines and proximal iodinated tyrosines.

While MD simulations offered meaningful insights into the evolution of the reaction, they could not provide a full description of the side chain rearrangement giving rise to T4. Thus, starting from representative MD frames of the iodinated forms of the model precursors (*i.e.* in which the two DITs are found in a stacking configuration), we outlined a minimal six-residue model system for the precursor and pursued DFT simulations to elucidate the mechanism of DIT coupling, providing its thermodynamic landscape (further details are reported in the Experimental Procedures section). T4 formation entails two events that can, in principle, be concomitant or sequential: the acceptor DIT O_ζ_ attacking the C_β_ of the donor DIT and dehydroalanine forming at the donor site *via* deprotonation of its C_α_ (Fig. 3*A*). Initially, we explored the feasibility of a sequential mechanism, in which an intermediate with a protonated C_α_ at the donor site is formed (Figs. 3, *A* and *B* and S8, ΔG1). We focused on the sole MBP-E374 case, and simulated scenarios involving either anionic, radical, or combined mechanisms for the DIT coupling process. These calculations revealed some fundamental insights regarding the nature of the coupling. They showed that the radical pathway is the most probable due to the notably lower free energy involved and that a sequential mechanism seems unlikely, suggesting a concerted route (Fig. 3, *A* and *C*, ΔG2). Indeed, although DFT does not capture the full extent of conformational flexibility, which may introduce some uncertainty in the calculated energies, the substantial energy differences we observe between the various mechanistic hypotheses allow us to confidently conclude that the process should be necessarily radical and concerted. Furthermore, it is not possible to discern between the acceptor or donor role, so the directionality of the coupling cannot be explained based on thermodynamic arguments. MD suggested that the two DITs may initially adopt an energetically preferred parallel stacking (Fig. 2). However, a more productive perpendicular stacking, where the hydroxyl group of one DIT directly points toward the γ-carbon of the other, facilitates product formation without requiring significant structural reorganization as in parallel stacking (Figs. 3*A* and S9). This perpendicular T-shaped disposition, achievable by populating different stable rotamers of one DIT, is likely an intermediate in T4 production. Intriguingly, transient perpendicular stacking is also observed between the two tyrosine residues and DITs in MD simulations (Fig. 2). We then quantified ΔG values associated with a concerted pathway, *i.e.*, directly leading to T4 at the acceptor tyrosine and to a dehydroalanine at the donor tyrosine, that indicated a favorable exergonic process for all the T4 precursor variants (Fig. 3*C*). Although energy differences along the series are quite small (and in some cases even below the accuracy limit of the method) we could remarkably notice that the estimated ΔG values are in line with the experimental trends of T4 yields, positioning E and D as most reactive (Figs. 1 and 3*C*). In addition, we could identify a possible stable coupling intermediate for the reaction and calculated relative ΔG values, for all the T4 precursor variants (Figs. S10 and S11). Our computational approach provided for the first time a thermodynamic picture of T4 formation, allowing the characterization of the mechanism and of the species likely involved in the lowest energy scenario.

### Early deprotonation of acceptor DIT determines directional synthesis of thyroxine

Starting from the *in silico* description of T4 synthesis in our model precursor, we sought to examine the role of the acidic residue preceding acceptor tyrosine in expediting T4 formation. Having verified that the DIT coupling step is likely radical in nature, we infer that this step should be initiated by the oxidation of the OH at the acceptor iodophenol ring. Since one-electron oxidation of phenolate is more favored than that of phenol (19) deprotonation of DIT assisted by the acidic residue would facilitate the formation of a radical acceptor site. Following up on previous studies (20) we then calculated that the relative pKa of tyrosine progressively decreases with the addition of iodine atoms on the aromatic ring and that at physiological pH the DIT is mostly found as de-hydrogenated (Figs. 3*D* and S12 for further details). This finding elucidates why T4 synthesis is enhanced in the presence of the acidic residues E and D, yet still occurs at lower rates in the mutants with residues K and S, as the oxidative state persists even without the preceding residue (Fig. 1, *C* and *D*). Moreover, DFT calculations showed that the deprotonation of DIT by the proximal E residue is only 4 kcal/mol, indicative of a favorable event. Our hypothesis is further confirmed by inspecting the MD trajectories of both E374 and D374. Here, we observe that the glutamate side chain maintains a non-negligible distance from the Oζ of the acceptor tyrosine. Therefore, the consensus Glu/Asp-Tyr consensus sequence can be considered a facilitating factor for its early deprotonation (Fig. 3*E*). Our findings are also in agreement with the previously reported pH dependency of the reaction, which is inhibited below pH 6, where dehydrogenation is less efficient (20). We propose that the initial oxidation at the phenyl OH group, rather than the coupling of DIT, is the limiting step for T4 synthesis. Indeed, when considering the coupling starting in the opposite direction, from the donor (Y341) instead of the acceptor (Y375), the calculated reaction energies and the ranking of the E, D, K, and S variants are similar. Therefore, we suggest that without the acidic residue on one of the two tyrosine, the reaction has no preferred directionality and T4 could be synthesized at both tyrosine positions conversely, the consensus Glu-Asp-Tyr determines T4 synthesis uniquely at the acknowledged acceptor positions within a protein precursor.

### Sequence conservation defines early thyroxine synthesis and release in TG hormonogenic sites

**Figure 4:**
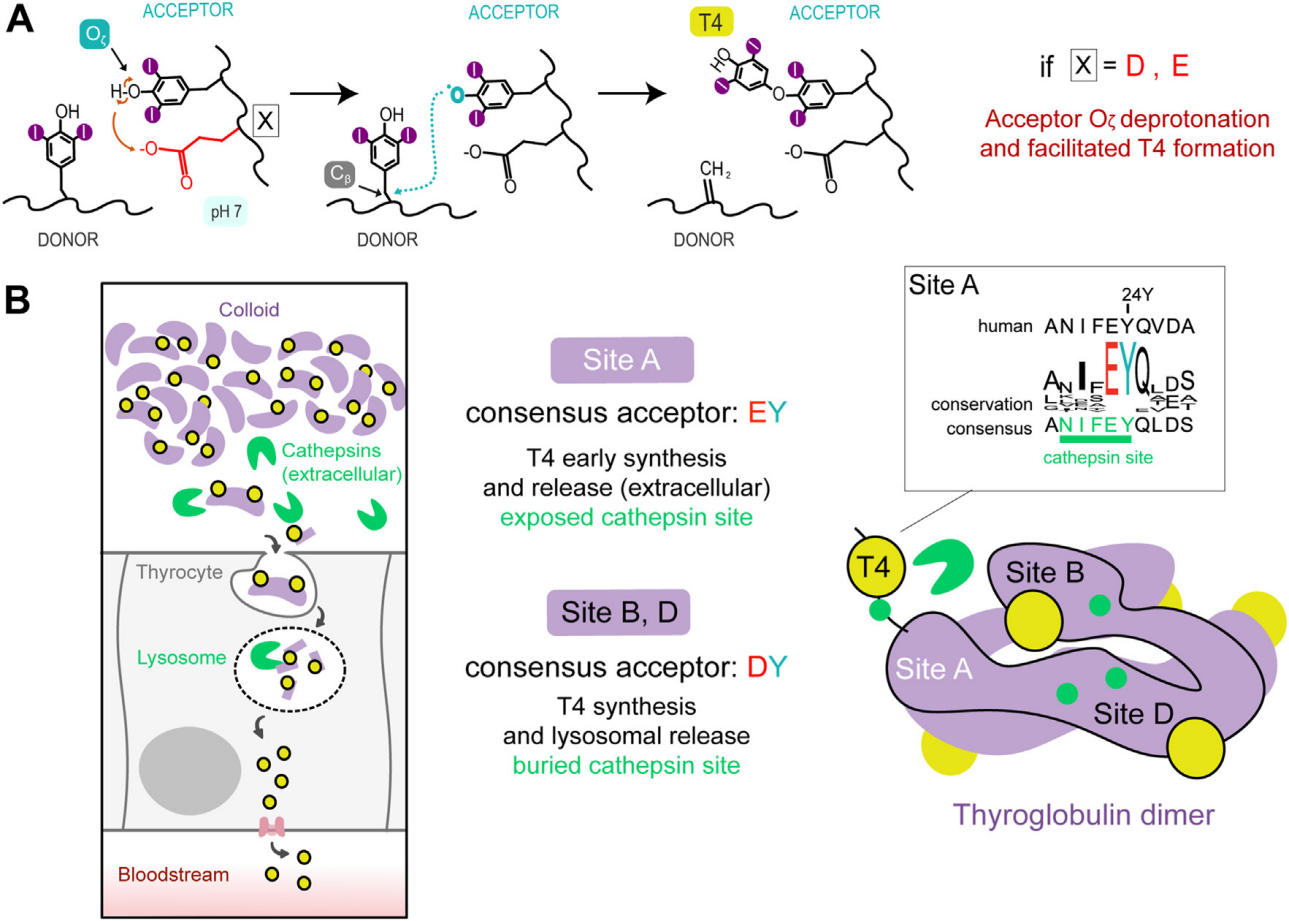
**Role of the consensus sequence Glu/Asp-Tyr in T4 formation within protein precursors.***A*, our model describes the role of an acidic residue (mainly glutamate) preceding the acceptor in facilitating deprotonation and driving directional T4 synthesis. *B*, our rationale for the coexistence of multiple T4 sites in TG: site A is flanked by an exposed cathepsin residue (early synthesis and release in the extracellular lumen of thyroid follicles), site B, D, and A (release in the lysosomes after endocytosis).

Our study on a model T4 precursor indicates that the presence of an acidic residue (preferably a glutamate) before one of the hormonogenic tyrosine, determines its deprotonation and hormone synthesis at the same position, thereby defining its role of iodophenyl acceptor tyrosine (Fig. 4*A*). In the natural TG precursor thyroid hormone synthesis is completed by proteolysis, and the directionality given by the acidic residue is crucial to position T4 where cleavage can be targeted by cathepsins (9, 17). In this scenario, at the highly conserved site A, the acceptor is preceded by a glutamate and it is flanked by an exposed cathepsin cleavage sequence NIFEY. Therefore, site A appears to provide both the quickest T4 synthesis and release, in agreement with an early hypothesis (21). Importantly, T4 release at site A can occur already in the extracellular lumen of the thyroid follicles, where TG maintains its three-dimensional structure (17). Conversely, we surmise that the late evolutionary sites B and D, where the acceptor is preceded by aspartate and the surrounding cathepsin sites are buried in the giant TG scaffold, provide a slightly slower synthesis and more gradual T4 release (Fig. 4*B*). In this case, the release can only be completed after endocytosis in the lysosomes, where TG structure is unfolded.

## Discussion

Thyroid hormone synthesis, with T4 being the main product, occurs *via* iodination of a handful of tyrosine pairs onto the complex and large fold of TG, a dimer of about 600 kDa in molecular weight. TG, made in large amounts in the thyroid, is then completely proteolyzed in the lysosomes to allow the release of the seven copies of the hormone (22). The overall process may appear inefficient, also considering that the same reaction can be recapitulated in much smaller scaffold proteins bearing flexible tyrosine pairs (10). On the other hand, thyroid hormone synthesis seems far from being arbitrary, as TG architecture is conserved across vertebrates (7) and early studies report intrinsic regulation of their yields at the different sites of their precursor TG (21, 23, 24, 25). In the structurally resolved sites A, B, D, T4 synthesis is directional, as the hormone is consistently found at only one position between the two active tyrosines, preceded by an acidic residue (by consensus, a glutamate in site A or an aspartate in site B and D) (26). Despite the structural characterization of iodinated mammalian TG and mutagenesis studies of the full protein (10, 12, 13), the implication of this sequence signature on thyroid hormone synthesis has been poorly understood. In this work, we investigated this aspect by implementing parallel *in vitro* synthesis and *in silico* quantitative modeling of a single-site model precursor, and by mutating the acidic residue preceding the acceptor. A structural comparison of the TG sites A, B, D (PDBID 6SCJ) with the hormonogenic MBP-E374 variant derived from MD simulations is reported in Fig. S14. For the first time, we provide dynamic and energetical information about the mechanism of T4 and general thyroid hormone synthesis. While both an anionic and radical mechanism for tyrosine coupling have been proposed for the tyrosine coupling (20), we show that the reaction is unambiguously radical in nature, and as such, favored by deprotonation of the di-iodo-tyrosine. Moreover, our calculations show a progressive decrease in pKa of the phenol group of tyrosine from 10 to 8.46 and 6.60 with the addition of one and two iodine atoms onto the aromatic ring, thereby facilitating the coupling reaction at physiological pH, in line with previous studies (20) (more details are reported in Fig. S12). Once iodotyrosines are formed, the coupling is symmetrical, *i.e.*., donor and acceptor are indistinguishable and T4 can be made at either position. When an acidic moiety precedes one of the tyrosines, its early deprotonation and iodination favor a directional attack toward the partner tyrosine. Hence, tyrosine iodination rather than coupling is the limiting step of the reaction and this explains why in TG the acceptor positions bearing T4 (26) are preceded by an acidic residue. It is therefore clear why di-iodo-tyrosine coupling and T4 synthesis are unfavoured at acidic pH (20). Our calculations show that in the case of the more extended glutamate side chain, deprotonation is more likely to occur as closer in space to the tyrosine Oζ with respect to an aspartate. These findings are coherent with previous reports describing the evolutionary ancient site A as the fastest T4 hormonogenic site (21). Overall, the rate of T4 synthesis is a combination of early formation onto the TG polypeptide chain and efficient proteolytic cleavage. Site A, within the exposed N-terminal peptide of TG, not only bears a glutamate before the acceptor tyr for earlier hormonogenesis, but also overlaps with a cathepsin cleavage site, promptly completing T4 release. We hypothesise that sites B and D, appearing later in evolution, are slower hormonogenic sites and do not overlap with cathepsin sites, allowing a late T4 release only at the lysosome level, upon protein unfolding. It must be noted that other isolated tyrosine residues are preceded by acidic residues, making them more prone to iodination, according to the same mechanism (14, 23). In conclusion, considering consensus sequence signatures, our mechanistic study indicates that the natural thyroid hormone precursor TG is not merely a pluri-iodine scavenger with few tyrosine pairs providing serendipitous T4 synthesis. TG sequence conservation holds significant importance for iodination and T4 synthesis and controlled release, suggesting potential coevolution of these functions. We think the importance of the Glu/Asp-Tyr sequence in thyroid hormone synthesis may also be a new route to identify thyroglobulin-like pre-vertebrate T4 precursors (27). Advanced DNA sequencing technologies allow the identification of many new mutations in the TG gene related to congenital hypothyroidism (28, 29, 30, 31, 32). Our study underlines that for the interpretation of mutation pathogenicity, it is crucial to take into account not only regions disrupting the overall protein fold but also flexible regions susceptible to dishormonogenesis (28, 30). To reveal these regions, it is key to continue developing more sophisticated experimental and computational tools to study iodination and hormone formation at the molecular level. Currently, it remains to be clarified how T4 synthesis happens in the symmetrical site C and how TG regulates T4 to T3 ratio and yields in different cellular conditions, such as post-translational modifications and iodine availability (16, 17). The present hybrid *in vitro* and *in silico* study of T4 synthesis represents a first step towards unveiling the ambiguous mechanistic understanding of thyroid hormone synthesis at an atomic level, building a framework to further study the regulation of hormone production in response to different physiological stimuli, as well as its alteration in disease (16, 30). It should be noted that we only reported T4 rate trends derived from iodination quenched at different time points, however, we still miss reliable kinetic tools to accurately follow every step of T4 synthesis. Our computational effort to elucidate the mechanism and stable intermediates of the T4 formation may inspire experimental biochemical methods to track them over time and derive reaction kinetic parameters. In addition, the engineered T4 protein precursor here described may be used to devise new strategies for *in vitro* T4 production for pharmaceutical usage, an alternative to classical organic chemistry synthesis (33).

## Experimental procedures

### Protein expression and purification

All hormonogenic MBP variants were expressed with an N-terminal 6x-histidine tag from the pHis17 vector in chemically competent *E. coli* Top10 cells. Cloning was performed using Q5 site-Directed Mutagenesis Kit (E0554) from New England Biolabs, ZymoPURE Plasmid Kits, and verified by Sanger sequencing (Eurofins).

For bacterial expression, MBP variants where residue 374 preceding the acceptor tyrosine was an E, D, S, or K were transformed into chemically competent C41(DE3) *E. coli cells*. Cells were grown in Luria Broth (LB) medium at 30 °C in the presence of 100 μg/ml ampicillin until cells reached an optical density at 600 nm (OD_600_) of about 0.8 to 1.0. Expression was induced with 1 mM IPTG overnight at 16 °C. Cells were harvested by centrifugation, resuspended in lysis buffer (50 mM Tris (pH 8), 200 mM NaCl supplemented with 20 mM imidazole, DNase I (MP Biomedicals), lysozyme (Sigma Aldrich) and protease inhibitors (Roche)), and lysed by sonication. The soluble cellular fraction was isolated by centrifugation and filtered with a 0.22 μm filter.

For affinity purification, clarified lysates were incubated with Ni-Sepharose HighPerformance resin beads (Cytiva), pre-equilibrated with 20 mM HEPES (pH 7.5), 150 mM NaCl (Buffer A). The resin was washed with Buffer A, supplemented with increasing concentrations of imidazole (20 mM, 50 mM), before elution with 500 mM imidazole. The eluting fractions containing histidine-tagged protein, as verified by SDS–PAGE analysis, were pooled and concentrated by ultrafiltration before further purification by size-exclusion chromatography. Proteins were further purified after the affinity purification steps using a Superdex 75 column (Cytiva). Pure protein fractions were concentrated and stored at −80 °C.

### *In vitro* enzymatic iodination assay

MBP at a final concentration of 0.49 g/l was added to 1 mM potassium iodide (KI, Sigma Aldrich), 6 mM glucose (Sigma Aldrich), 0.002 g/l glucose oxidase (Sigma Aldrich), and 0.003 g/l lactoperoxidase (Sigma Aldrich). The assay was conducted in 50 mM Tris (pH 8.0), 200 mM NaCl buffer. Incubations were initiated with the addition of lactoperoxidase (LPO), incubated for 1 hour at 37 °C. For time course studies, the reaction was inactivated after incubation by heating at 95 °C for 2 min.

### Immunoblots

For dot blot, samples (about 1.5 μg) were spotted onto nitrocellulose membranes. The membrane was blocked in TBST buffer (10 mM Tris-HCl (pH 7.4), 150 mM NaCl, 0.1% v/v Tween 20) containing 2.5% bovine-serum-albumin (BSA) for 1 hour at room temperature. The appropriate working range for antibodies was optimized starting from vendor dilution recommendations. Membranes were incubated with two primary antibodies of different host species in 2.5% BSA TBST as follows: Antithyroglobulin (1:1000, Abcam, rabbit), Anti-6X His tag (1:1000, Abcam, rabbit), Antithyroxine (1:1000, Santa Cruz Biosciences, mouse). Primary antibodies were incubated separately; the first was incubated overnight at 4 °C followed by extensive washes in TBST, before a second primary antibody incubation of 2 hours at room temperature. After further washes in TBST, membranes were incubated with antirabbit Alexa-Fluor-linked secondary antibody (1:10,000, IRDye 680RD, LI-COR Biosciences) or anti-mouse Alexa-Fluor-linked secondary antibody (1:10,000, IRDye 800CW, LI-COR Biosciences). Immunoreactive proteins were detected by infrared fluorescence (692 nm and 792 nm) using the Odyssey Imager from LI-COR Biosciences.

### ELISA hormone quantification

The yield of T4 was quantified using the commercially available enzyme-linked immunosorbent assay (ELISA) originally designed for detecting hormone products in blood serum (Abcam, ab178664). Following iodinated sample preparation, pronase protease mix (Roche) was added at about 0.0025 g/l to digest all enzymes, and to release thyroid hormones for 40 min at 40 °C. This was followed by heat inactivation for 15 min at 95 °C. Bovine serum albumin at a concentration of 60 g/l was added as an essential blocking agent. The samples were then transferred to ELISA plates for detection, following the manufacturer’s instructions. Using the manufacturer's calibration curve, absorbance at 450 nm was converted into T4 concentration. Each measurement was performed at least three times independently and reported the average and standard deviation values.

### Additional mass spectrometry processing

For analysis of post-translational modifications (methionine oxidation, MIT, DIT, T4 and dehydroalanine on tyrosine) excised bands containing about 10 μg protein were submitted to Cogentech SRL. There, samples were digested by trypsin or endoproteinase Glu-C and extracted by StageTip C18. Analysis was done over a 75 min gradient on nLC-ESI-MS/MS. Results were analyzed by the manufacturer using ProteomeDiscoverer (version 1.4), Mascot, and Scaffold. This analysis performed on iodinated MBP was performed by the IFOM MS facility Cogentech (Milano, IT).

### Molecular dynamics studies

#### Starting structures

The starting structure of maltose binding protein (MBP) has been obtained from the RCSB Protein Data Bank (PDB ID: 1ANF) and manually edited in PyMOL (https://pymol.org/) at its C-terminus in order to generate four extended β-sheet configurations (*i.e.*, Φ = −120°, Ψ = 120°) of the engineered tails corresponding to sequences S_371_GSXYS_376_ (SGSXYS for short within the rest of the text), with X = D,E,K,S (34). The corresponding structures have been therefore named 1anf-SGSDYS, 1anf-SGSEYS, 1anf-SGSKYS, and 1anf-SGSSYS, respectively, and have been eventually edited to replace the tyrosine residue 375 with a.

3,5-di-iodotyrosine, *i.e.*, with iodine atoms in Positions 3 and 5 (corresponding to Hε1/2 positions) on the aromatic ring (see Supporting information). Every structure has been manually edited to replace tyrosine residues (TYR) with 3,5-di-iodotyrosine residues (TYD) for Molecular Dynamics (MD) simulations.

#### Topology of 3,5-di-iodotyrosine

The parametrization for Molecular Dynamics simulations of 3.5-di-iodotyrosine has been realized by means of Self Consistent Field (SCF) restricted Hartree–Fock (RHF) calculations performed in NWChem as implemented in the BiKi suite (35, 36). The molecular wavefunction and the corresponding electrostatic potential of the tripeptide A-TYD-A (with TYD = 3,5-di-iodotyrosine) have been computed, *i.e.*, a system that minimizes the bias of neighbors’ steric hindrance and charge on the variability of TYD electronic structure. A 6-31G∗ basis set has been used for all the atoms included in the structure (37) except for the 6 to 311G∗ basis set adopted for iodine atoms, with a total amount of 540 basis functions. The quantum mechanical (QM) electrostatic potential has been computed using 7822 grid points spaced by 0.5 A, a probe radius of 0.7 A and an atomic radius factor of 1. The RESP (Restrained ElectroStatic Potential) algorithm has been applied to the quantum mechanically (QM) calculated molecular electrostatic potential (ESP) at molecular surfaces using an atom-centered point charge model (38). The classical mechanical parameters for TYD have been generated using the generalized Amber force field (GAFF), integrated in the format of a standard residue topology in the Amber 99S-ILDN force field (39). The translation for the GROMACS package for molecular dynamics simulations has been performed using ACEPYPE (40, 41).

The topology in GROMACS format is reported in the Supporting information. The impact of the introduction of non-standard amino acid force-field parameters has been verified by 3 × 100 ns MD simulations (with the same setup mentioned above) of the model peptide AYAYA with tyrosines and with di-iodotyrosines and monitoring the contacts between tyrosines or di-iodotyrosines sidechains and the torsional sidechain dynamics, *i.e.*, Chi1 angle (Supporting Information).

#### Molecular dynamics

All the systems considered for the present work (*i.e.*, 1anf-SGSDYS, 1anf-SGSEYS, 1anfSGSKYS, and 1anf-SGSSYS) have been parameterized in GROMACS 2020.1 using the AMBER99-SB ILDN force field both in the non-iodinated form and in the iodinated form (39, 40). A parallelepipedal solvent box has been created around the protein, solvated with TIP3P water molecules (∼22,000). The net charge has been neutralized (with Na^+^ ions) and the electrostatics treated with the Particle Mesh Ewald (PME) scheme (42, 43). After minimization with the steepest descent method (convergence:100 kJ mol^–1^ nm^–1^), the system was equilibrated (with isotropic positional restraints on protein heavy atoms, k = 1000 kJ mol^–1^ nm^–2^) for 2 ns in the NPT ensemble with *p* = 1 atm and T = 300 K, then for 2 ns in the NVT ensemble at T = 300 K (44). Eventually, we performed 100 ns of simulation for each system in the NVT ensemble employing a timestep of 2 fs and constraining all covalent bond lengths with the LINCS algorithm (45). Every simulation has been replicated three times to increase the sampling statistics. The analyses have been performed using the GROMACS internal programs and routines with the support of in-house code and visually inspected using Visual Molecular Dynamics (VMD) (46). The molecular graphics have been realized using Gnuplot (http://gnuplot.info), python matplotlib, and PyMOL (https://pymol.org/) (47).

### DFT calculations

Quantum mechanical calculations were performed in the framework of Density Functional Theory (DFT), starting from representative frames of MD simulations on the iodinated MBP. More in detail, a cluster approach was chosen, by building up minimal models comprising the two DIT residues and their surroundings, for a total of six residues per model (X374-DIT375-S376 and W340-DIT341-A342, with X = D, E, S or K). Specific and selected alpha carbon positions (*i.e.* residues X374, W340, and A342) were fixed at their initial position, to avoid artifacts arising from the lack of the whole protein.

Geometry optimizations were carried out with the TURBOMOLE 7.4.1 suite of programs (37) and the pure BP86 functional (48, 49) was used in conjunction with a triple-ζ def2-TZVP basis set (50) (def2TZVP/ECP for iodine (51)). To exclude functional dependency of the calculated energy variations, we performed single-point calculations with the hybrid functional B3LYP (48, 52) on BP86-optimized geometries for a selected subset of structures/reactions (Fig. S13). B3LYP calculated energy variations differ by less than 1 kcal/mol on average (at the limit of DFT accuracy) from BP86 ones, indicating the absence of functional dependency on the calculated mechanisms. London dispersion forces were explicitly accounted for by applying the D4 correction scheme (53, 54) during optimizations. The Conductor-like Screening Model (COSMO) for implicit solvation was used for all calculations and, given the high solvent exposure of the portion of the protein under investigation, an ε = 80 (water) was set (55). The Resolution-of-Identity (RI) technique allowed to speed up all the structure optimizations (56). Natural population analysis (NPA) was carried out to calculate atomic charges for the structures of interest, performing single-point calculations with the B3LYP functional on BP86-optimized geometries. The S = 0 solution for diradical species has been calculated with the BrokenSymmetry (BS) approach, which allows the treatment of antiferromagnetic couplings within the monodeterminantal scenario of DFT (57).

### Sequence alignments

To perform TG conservation analysis in vertebrates (7) and avoid redundancy in higher vertebrates, we considered 3 TG sequences for each group (fishes, amphibians, birds, reptiles, mammals) with high quality and similar length (about 2700 amino acidic residues). We then performed sequence alignment and calculated conservation using MSA probs and Jalview software (Fig. S1) (58, 59).

## Data availability

All data are contained in the manuscript and supporting information file.

## Supporting information

This article contains supporting information.

## Conflict of interest

The authors declare that they have no conflicts of interest with the contents of this article.

## Supporting information

Supplementary Information

Supplementary Vedio
