# Supplementary material for: A conserved acidic residue drives thyroxine synthesis within thyroglobulin and other protein precursors
Source: J Biol Chem. 2024 Nov 26;301(1):108026. doi: 10.1016/j.jbc.2024.108026 (PMC11730217; doi:10.1016/j.jbc.2024.108026)
Supplement: Supplementary Information [file mmc1.docx]

**Supporting information**

**Figure S1 – Sequence alignment for TG hormonogenic sites.** The cartoon on the left depicts the location of the hormonogenic sites within the TG dimer: Site A, B, D are structured while Site C is flexible and structurally unresolved. On the right the sequence alignments within each site around the acceptor tyrosine, showing a consensus E/D-Y sequence for the structured sites.


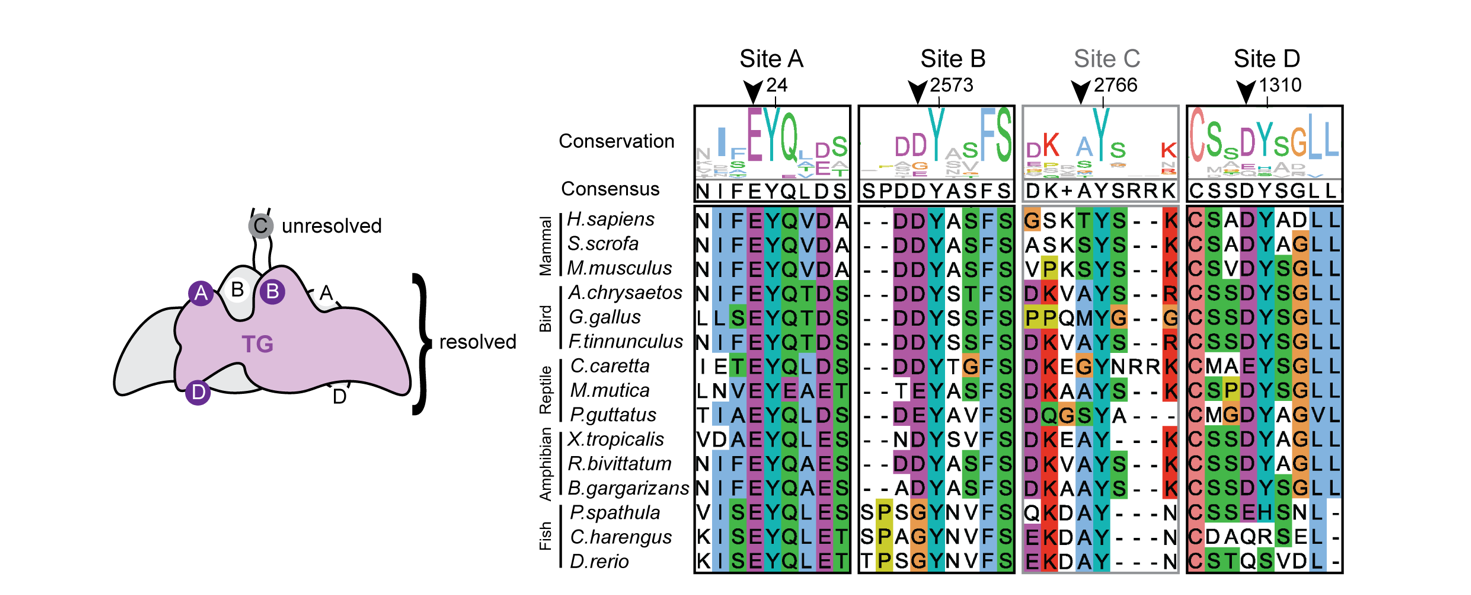


**Figure S2: Mass spectrometry analysis of iodinated MBP.** Full sequence of the engineered MBP thyroid hormone precursor and mass spectrometry analysis of the iodinated protein, showing a single acceptor site T4 at residue 375 and a unique Dehydroalanine (Dha) at residue . Therefore, the engineered MBP is a single site thyroid hormone precursor.


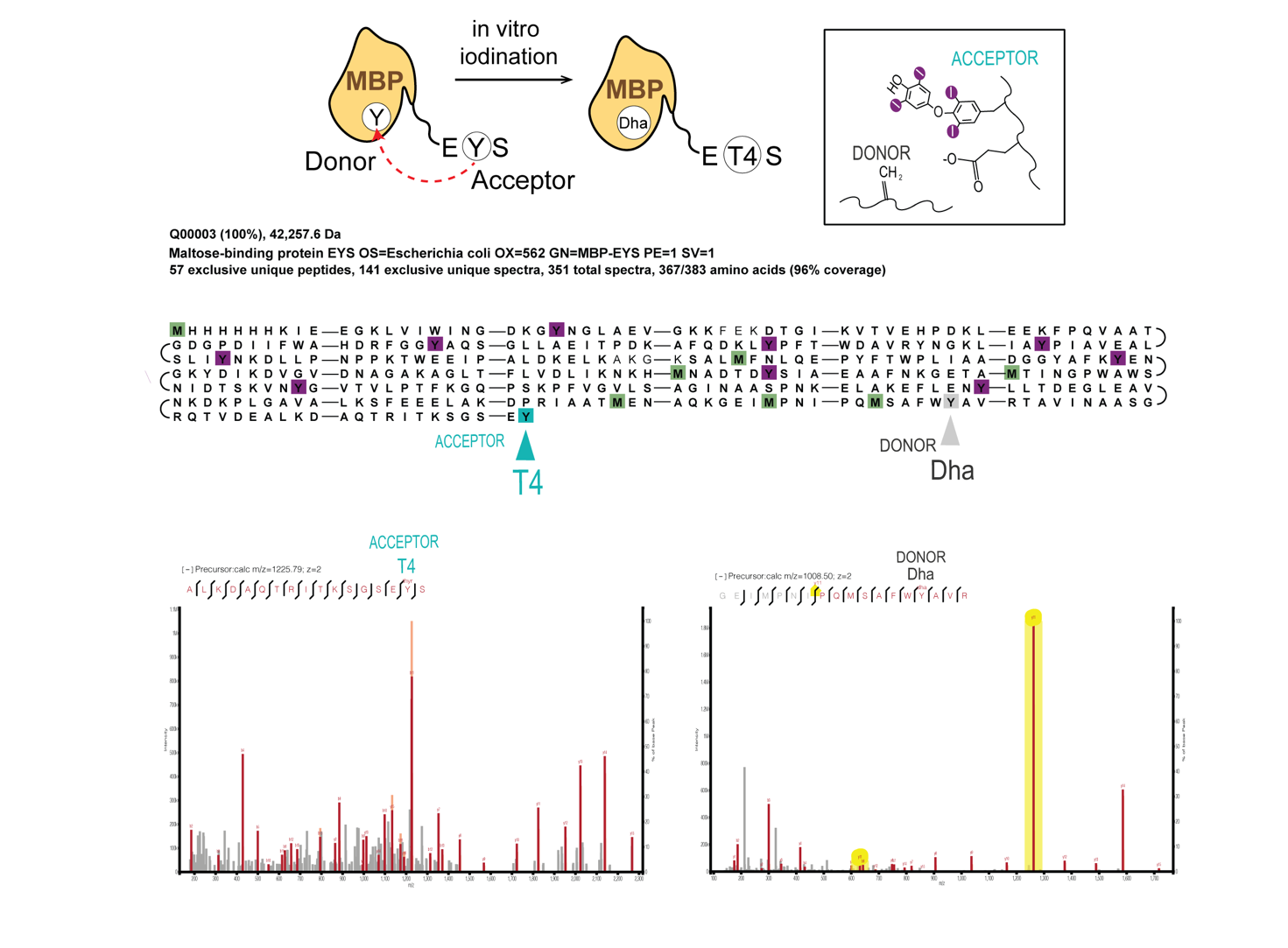


**Figure S3- Dot blot annotations.** Annotations of dot blot membranes spottend with iodinated MBP constructs, for the relative quantification of T4 content


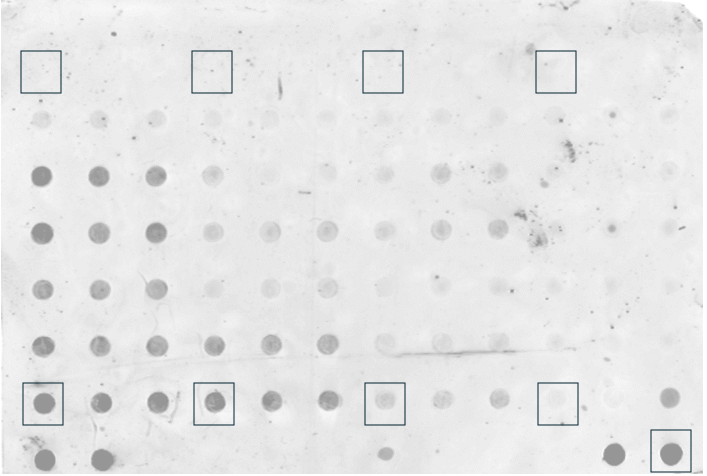


Iodination of MBP after 10 min incubation with the iodination reaction mix.

x= sample; Ctrl1 = T4 conjugated to BSA; Ctrl2=iodinated TG

| Iodine concentration | Construct: | MBP DYS | | | MBP EYS | | | MBP KYS | | | **corner cut*  MBP SYS | | |
| --- | --- | --- | --- | --- | --- | --- | --- | --- | --- | --- | --- | --- | --- |
| (uM) |  | 1 | 2 | 3 | 4 | 5 | 6 | 7 | 8 | 9 | 10 | 11 | 12 |
| 0 | a | x | x | x | x | x | x | x | x | x | x | x | x |
| 1 | b | x | x | x | x | x | x | x | x | x | x | x | x |
| 10 | c | x | x | x | x | x | x | x | x | x | x | x | x |
| 50 | d | x | x | x | x | x | x | x | x | x | x | x | x |
| 200 | e | x | x | x | x | x | x | x | x | x | x | x | x |
| 500 | f | x | x | x | x | x | x | x | x | x | x | x | x |
| 1000 | g | x | x | x | x | x | x | x | x | x | x | x | x |
|  | H | Ctrl  1 | Ctrl  2 |  |  |  |  |  |  |  |  | Ctrl  1 | Ctrl  2 |


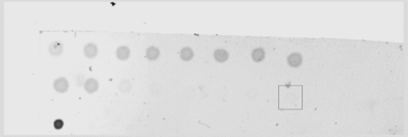


Iodine titration of recombinant thyroglobulin reference dot blot. 10 minute incubation.

Ctrl1 = T4 conjugated to BSA.

| KI conc. (uM) | 1 | 2 | 3 | 4 | 5 | 6 | 7 | 8 |
| --- | --- | --- | --- | --- | --- | --- | --- | --- |
| A | 1000 | 500 | 250 | 125 | 62.5 | 31.25 | 15.625 | 7.8125 |
| B | 3.90 | 1.95 | 0.98 | 0.49 | 0.24 | 0.12 | 0.061 | 0 |
| C | Ctrl1 |  |  |  |  |  |  |  |

**Figure S4: Nomenclature of DIT molecules**. 5-diiodotyrosine structure are depicted in 2D. Both standard naming of the atomic positions and numbering according to the classical mechanics topology (see Supplementary Table 1) are reported.


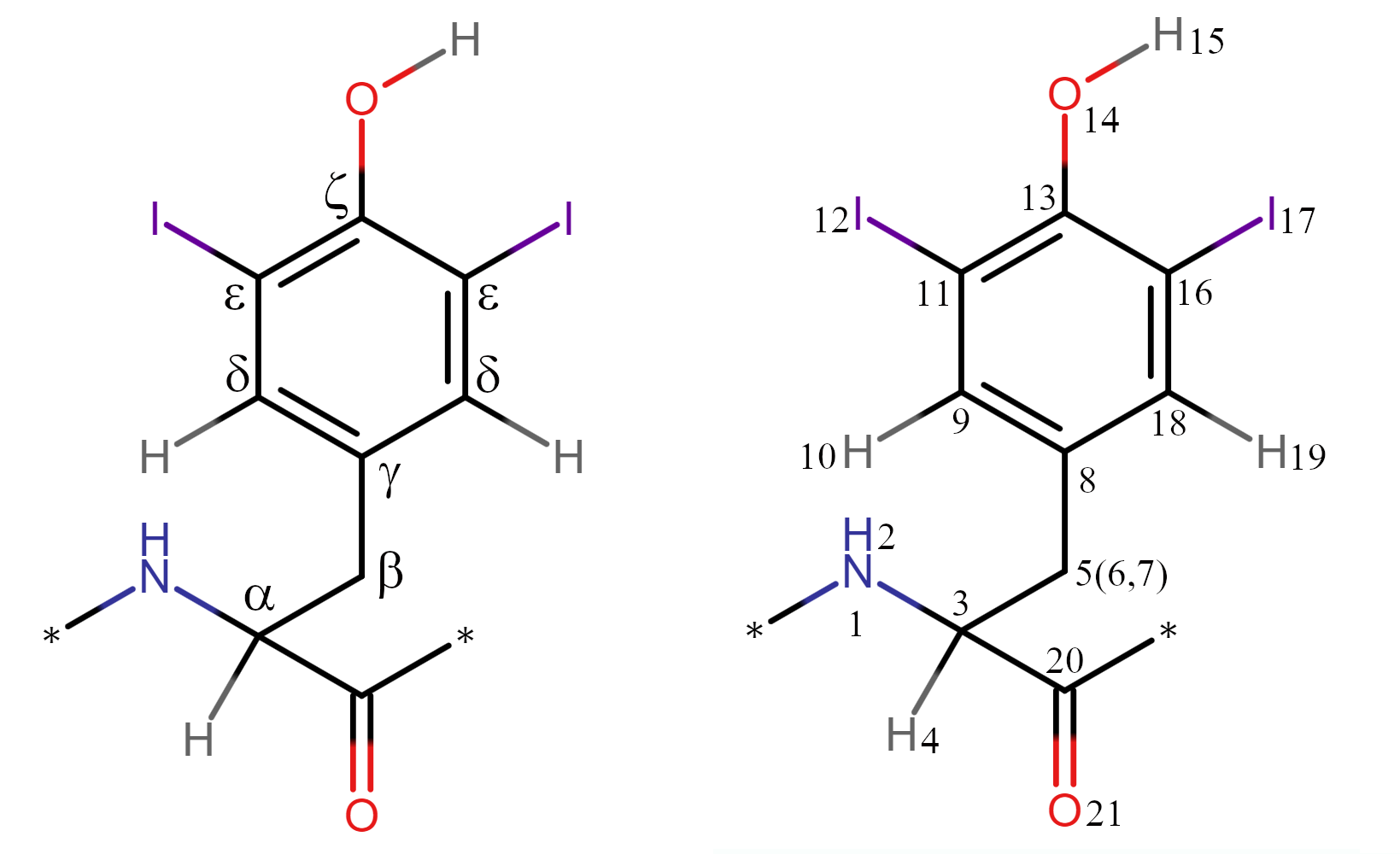


**Figure S5: Molecular dynamics trajectories for not iodinated MBP tyrosine pairs**

Cβ356 - Oζ374 (upper dataset) and Cβ356 - Cβ374 (lower dataset) distances for non-iodinated structures with distal Tyr 356 - Tyr 374 configuration as a cumulative function of time (i.e., 3 x 100 ns concatenated trajectories).


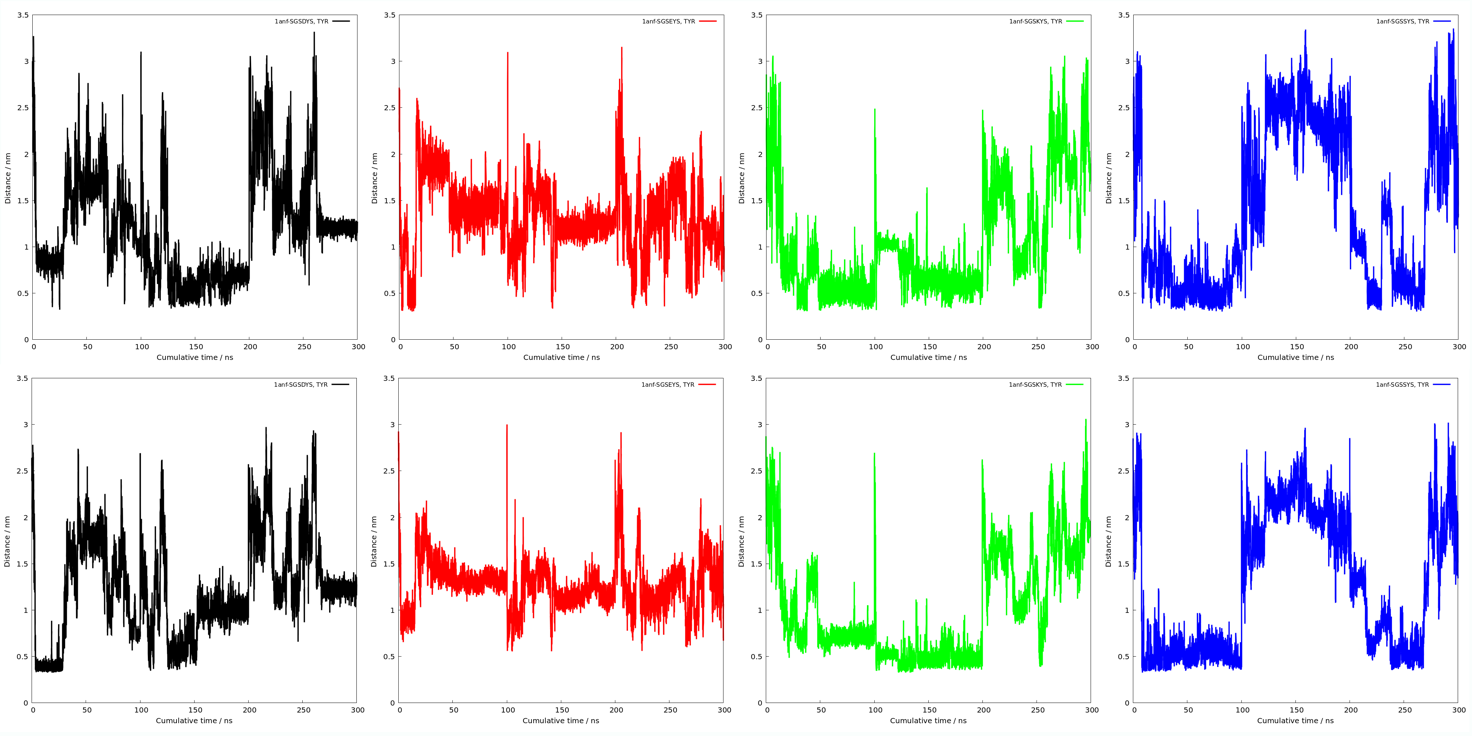


**Figure S6: Molecular dynamics trajectories for distally iodinated MBP tyrosine pairs**

Cβ356 - Oζ374 (upper dataset) and Cβ356 - Cβ374 (lower dataset) distances for iodinated structures with distal Tyr 374 – Tyr 356 configuration as a cumulative function of time (i.e., 3 x 100 ns concatenated trajectories).

**
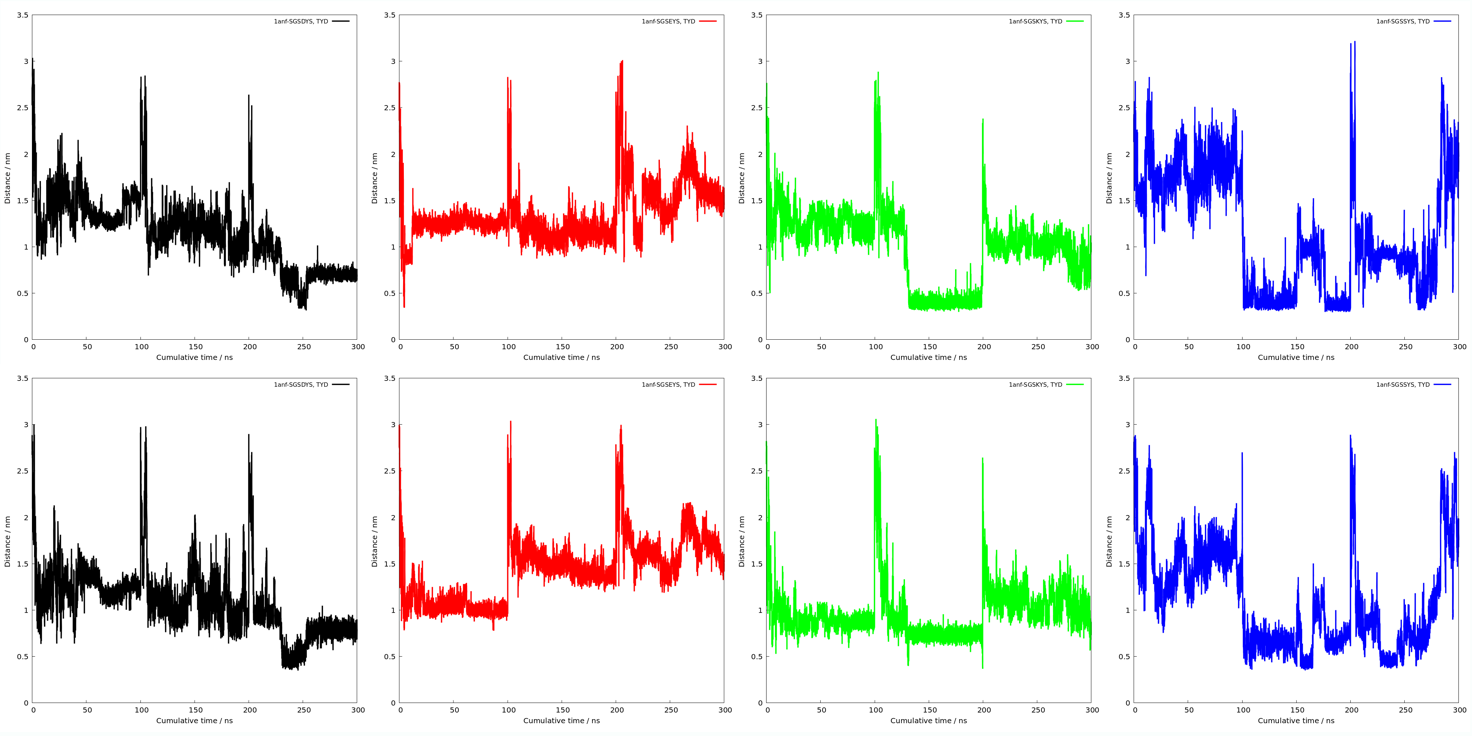
**

**Figure S7: Molecular dynamics trajectories for proximally iodinated MBP tyrosine pairs**

Cβ356 - Oζ374 (upper dataset) and Cβ356 - Cβ374 (lower dataset) distances for iodinated structures with proximal Tyr 374 – Tyr 356 configuration as a cumulative function of time (i.e., 3 x 100 ns concatenated trajectories).


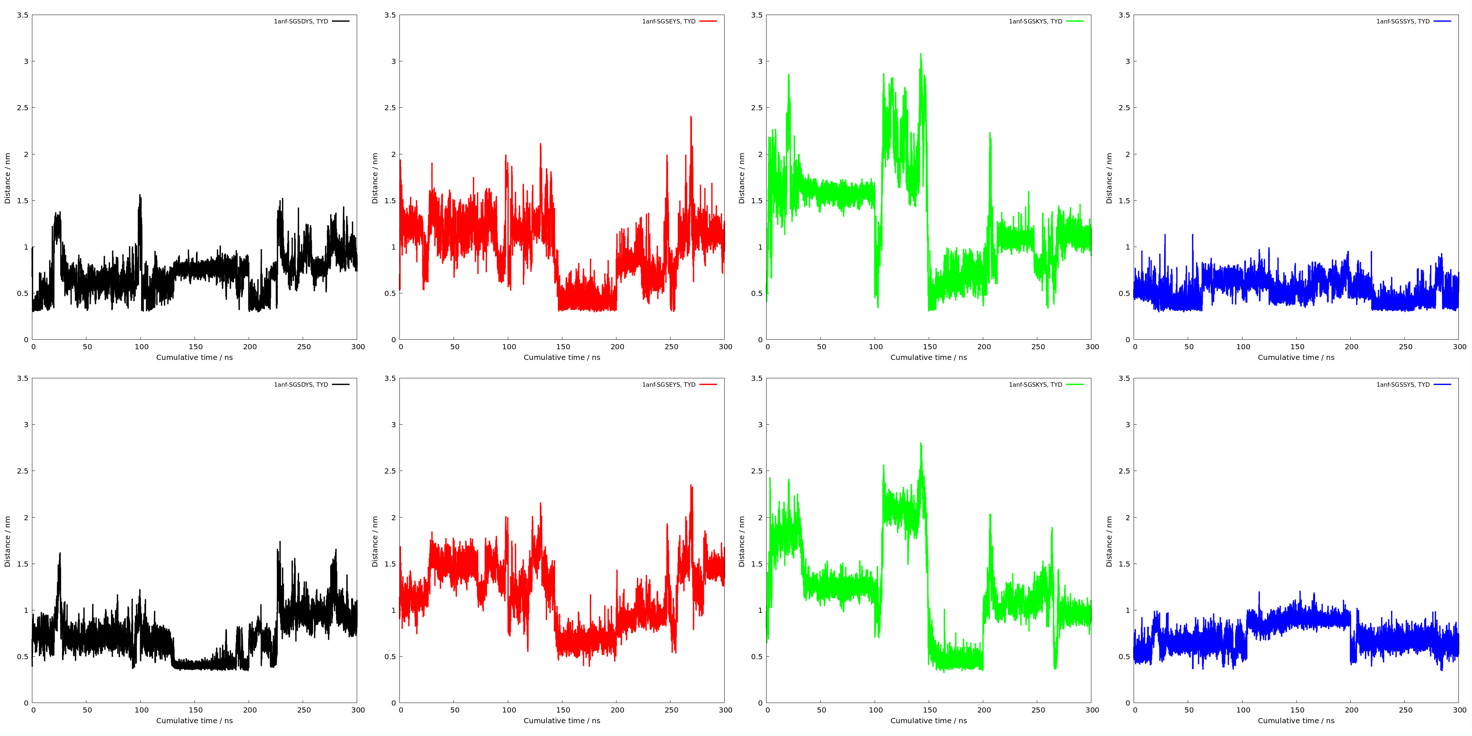


**Table S1: GROMACS Topology file for 3,5-diodotyrosine**

| [ TYD ] | G |  | |  | |  | | | |  | | |  | | |  | | |
| --- | --- | --- | --- | --- | --- | --- | --- | --- | --- | --- | --- | --- | --- | --- | --- | --- | --- | --- |
|  |  |  | |  | |  | | | |  | | |  | | |  | | |
| [ atomtypes ] |  |  | |  | |  | | | |  | | |  | | |  | | |
| IY | 53 | 126.9 | | 0 | | A | | | | 0.418722 | | | 167.360 | | |  | | |
|  |  |  | |  | |  | | | |  | | |  | | |  | | |
| [bondtypes] |  |  | |  | |  | | | |  | | |  | | |  | | |
| ; distances: column 4; unit = nm | |  | |  | |  | | | |  | | |  | | |  | | |
| ; forces: column 5; unit = kJ/(mol nm) | |  | |  | |  | | | |  | | |  | | |  | | |
| CA | IY | 1 | | 0.214 | | 197092.800 | | | |  | | |  | | |  | | |
|  |  |  | |  | |  | | | |  | | |  | | |  | | |
| [ angletypes ] |  |  | |  | |  | | | |  | | |  | | |  | | |
| ; angle: column 5; unit = ° (sexagesimal degrees) | | | | |  | |  | |  | | |  | | |  | | |  |
| ; forces: column 6; unit = kJ/(mol °) | |  | |  | |  | | | |  | | |  | | |  | | |
| IY | CA | CA | | 1 | | 120.000 | | | | 418.400 | | |  | | |  | | |
| IY | CA | C | | 1 | | 120.000 | | | | 418.400 | | |  | | |  | | |
|  |  |  | |  | |  | | | |  | | |  | | |  | | |
| [ dihedraltypes ] |  |  | |  | |  | | | |  | | |  | | |  | | |
| ; angle: column 6; unit = ° (sexagesimal degrees) | | |  | | | |  |  | | |  | | |  | | |  |  |
| ; forces: column 7; unit = kJ/(mol °) | |  | |  | |  | | | |  | | |  | | |  | | |
| X | X | CA | | IY | | 4 | | | | 180.000 | | | 15.180 | | | 2 | | |
| C | CA | CA | | IY | | 9 | | | | 180.000 | | | 15.180 | | | 2 | | |
|  |  |  | |  | |  | | | |  | | |  | | |  | | |
| [ atoms ] |  |  | |  | |  | | | |  | | |  | | |  | | |
| ; atom names: column 1; atom types (GAFF/GROMACS): column 2 | | | | | | |  |  | | |  | | |  | | |  |  |
| ; charges: column 3; unit = adimensional | |  | |  | |  | | | |  | | |  | | |  | | |
| N | N | -0.55 | | 1 | |  | | | |  | | |  | | |  | | |
| H | H | 0.32 | | 2 | |  | | | |  | | |  | | |  | | |
| CA | CT | 0.04 | | 3 | |  | | | |  | | |  | | |  | | |
| HA | H1 | 0.1 | | 4 | |  | | | |  | | |  | | |  | | |
| CB | CT | -0.04 | | 5 | |  | | | |  | | |  | | |  | | |
| HB1 | HC | 0.07 | | 6 | |  | | | |  | | |  | | |  | | |
| HB2 | HC | 0.07 | | 7 | |  | | | |  | | |  | | |  | | |
| CG | CA | -0.12 | | 8 | |  | | | |  | | |  | | |  | | |
| CD1 | CA | -0.04 | | 9 | |  | | | |  | | |  | | |  | | |
| HD1 | HA | 0.15 | | 10 | |  | | | |  | | |  | | |  | | |
| CE1 | CA | -0.04 | | 11 | |  | | | |  | | |  | | |  | | |
| IY1 | IY | -0.12 | | 12 | |  | | | |  | | |  | | |  | | |
| CZ | C | 0.2 | | 13 | |  | | | |  | | |  | | |  | | |
| OH | OH | -0.48 | | 14 | |  | | | |  | | |  | | |  | | |
| HH | HO | 0.44 | | 15 | |  | | | |  | | |  | | |  | | |
| CE2 | CA | -0.04 | | 16 | |  | | | |  | | |  | | |  | | |
| IY2 | IY | -0.12 | | 17 | |  | | | |  | | |  | | |  | | |
| CD2 | CA | -0.04 | | 18 | |  | | | |  | | |  | | |  | | |
| HD2 | HA | 0.15 | | 19 | |  | | | |  | | |  | | |  | | |
| C | C | 0.65 | | 20 | |  | | | |  | | |  | | |  | | |
| O | O | -0.61 | | 21 | |  | | | |  | | |  | | |  | | |
|  |  |  | |  | |  | | | |  | | |  | | |  | | |
| [ bonds ] |  |  | |  | |  | | | |  | | |  | | |  | | |
| N | H |  | |  | |  | | | |  | | |  | | |  | | |
| N | CA |  | |  | |  | | | |  | | |  | | |  | | |
| CA | HA |  | |  | |  | | | |  | | |  | | |  | | |
| CA | CB |  | |  | |  | | | |  | | |  | | |  | | |
| CA | C |  | |  | |  | | | |  | | |  | | |  | | |
| CB | HB1 |  | |  | |  | | | |  | | |  | | |  | | |
| CB | HB2 |  | |  | |  | | | |  | | |  | | |  | | |
| CB | CG |  | |  | |  | | | |  | | |  | | |  | | |
| CG | CD1 |  | |  | |  | | | |  | | |  | | |  | | |
| CG | CD2 |  | |  | |  | | | |  | | |  | | |  | | |
| CD1 | HD1 |  | |  | |  | | | |  | | |  | | |  | | |
| CD1 | CE1 |  | |  | |  | | | |  | | |  | | |  | | |
| CE1 | IY1 |  | |  | |  | | | |  | | |  | | |  | | |
| CE1 | CZ |  | |  | |  | | | |  | | |  | | |  | | |
| CZ | OH |  | |  | |  | | | |  | | |  | | |  | | |
| CZ | CE2 |  | |  | |  | | | |  | | |  | | |  | | |
| OH | HH |  | |  | |  | | | |  | | |  | | |  | | |
| CE2 | IY2 |  | |  | |  | | | |  | | |  | | |  | | |
| CE2 | CD2 |  | |  | |  | | | |  | | |  | | |  | | |
| CD2 | HD2 |  | |  | |  | | | |  | | |  | | |  | | |
| C | O |  | |  | |  | | | |  | | |  | | |  | | |
| -C | N |  | |  | |  | | | |  | | |  | | |  | | |
|  |  |  | |  | |  | | | |  | | |  | | |  | | |
| [ impropers ] |  |  | |  | |  | | | |  | | |  | | |  | | |
| -C | CA | N | | H | |  | | | |  | | |  | | |  | | |
| CA | +N | C | | O | |  | | | |  | | |  | | |  | | |
| CG | CE2 | CD2 | | HD2 | |  | | | |  | | |  | | |  | | |
| CZ | CD2 | CE2 | | IY2 | |  | | | |  | | |  | | |  | | |
| CD1 | CZ | CE1 | | IY1 | |  | | | |  | | |  | | |  | | |
| CG | CE1 | CD1 | | HD1 | |  | | | |  | | |  | | |  | | |
| CD1 | CD2 | CG | | CB | |  | | | |  | | |  | | |  | | |
| CE1 | CE2 | CZ | | OH | |  | | | |  | | |  | | |  | | |

**Figure S8: Detailed hypothesis of mechanism for T4 formation.** Mechanistic hypotheses unraveled by DFT for a sequential mechanism, entailing the formation of an intermediate with a Cα-H bond at the donor site (here referred to as INT1). The various pathways have been envisioned considering oxidation/protonation events occurring at different stages. Indeed, oxidation can occur before the attack of one DIT towards the other, supporting a radical mechanism, or it may occur after the attack which, in this last case, would follow an anionic mechanism. ΔG1 values have been calculated focusing on a single T4 precursor (EYS) considering DIT375 acting as both acceptor and donor, and are collected in Figure 3 of the manuscript. These calculations allowed us to rule out INT1 formation as part of the coupling mechanism and determine the attack's radical nature.


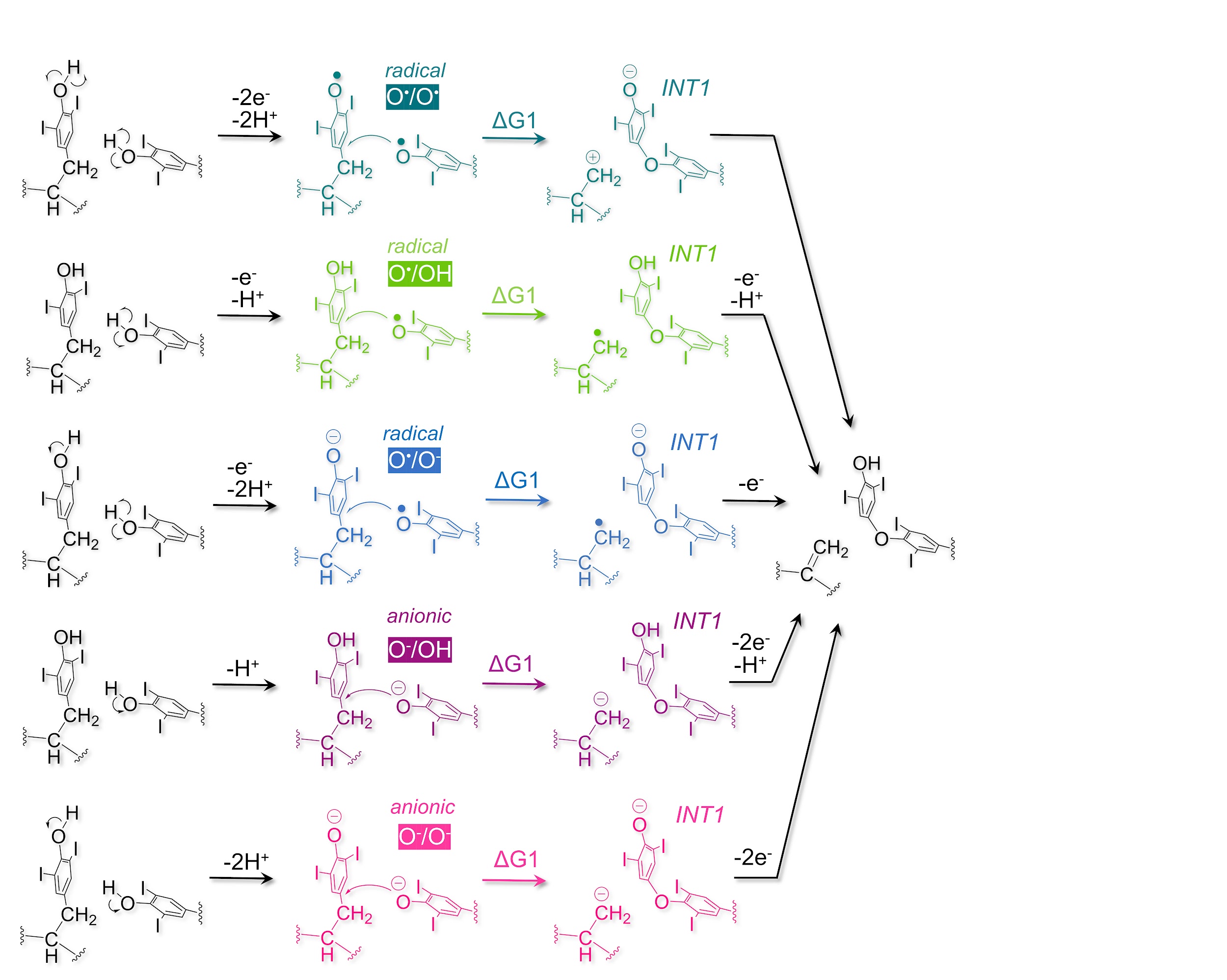


**Figure S9: Optimized structures of parallel and perpendicular stacked DITs, obtained for the EYS T4 precursor.** In general, two perpendicular dispositions can be formed starting from parallel DITs, by rotating alternatively one of the two DITs side chains. According to the perpendicular stacking disposition formed, one DIT acts as acceptor (A) or as donor (D) in the coupling mechanism. Black values refer to the relative free energy (in kcal/mol) between perpendicular stacked DITs and parallel ones, considering different redox/ionic states of the two side chains and obtained for EYS. Two sets of perpendicularly stacked DITs are shown, for DIT 375 acting as acceptor (orange arrow, ΔG 〜 8.5 kcal/mol on average) or donor (purple arrow, 〜 10.5 kcal/mol on average). *In the case of biradical states, the BS singlet (S=0) solution is considered (more stable than the S=1 one).


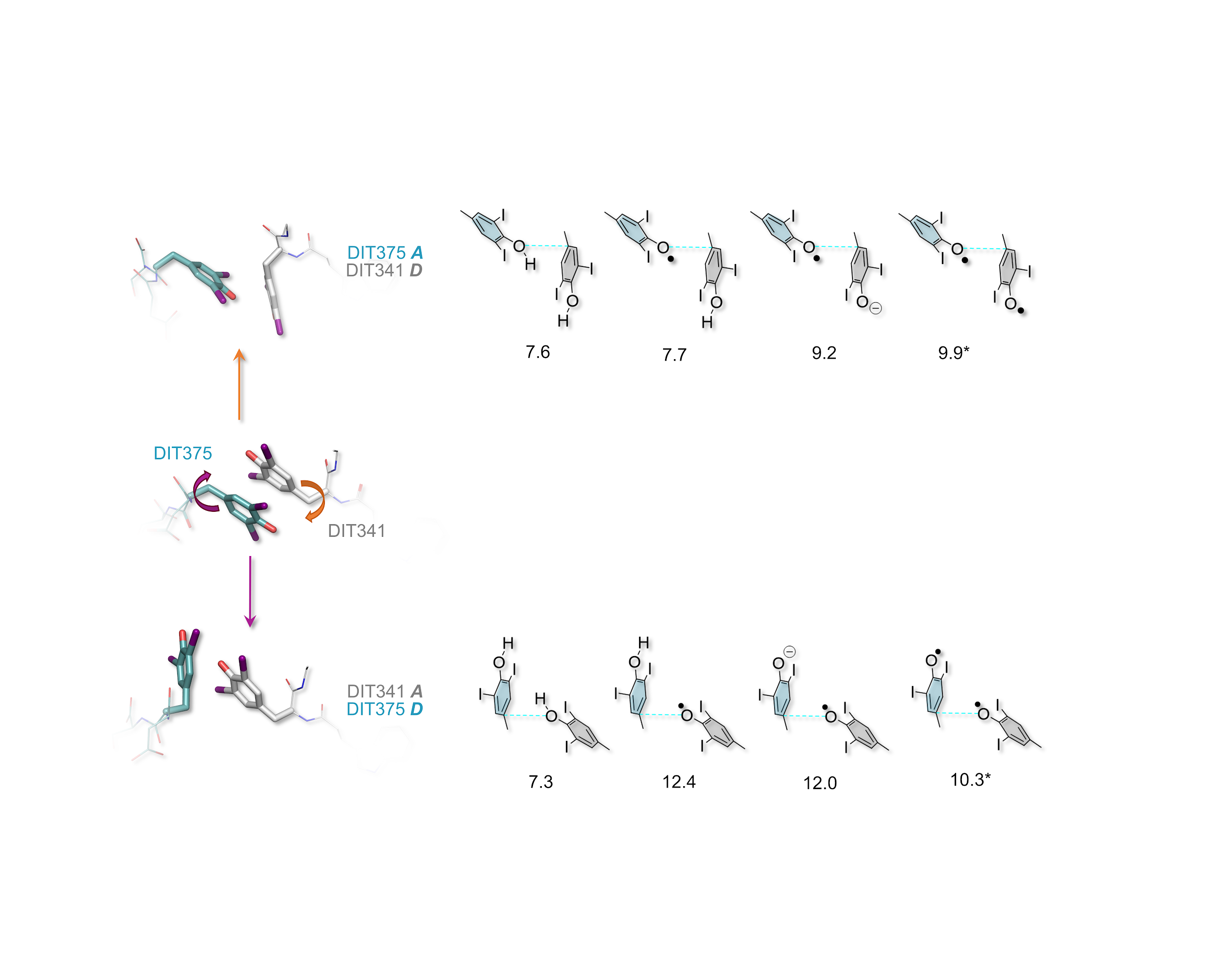


**Figure S10: Calculated lowest energy mechanism for DITs coupling.** Free energy values (in kcal/mol) refer to doubly oxidized systems in a singlet (BS S=0) state, taken as an example. This situation is compatible with a very likely scenario in which a radical is formed at the acceptor site (triggered by deprotonation), driving the coupling direction, and a second oxidation occurs concomitantly with the attack. This mechanistic route is the most favorable since it implies the coupling of the two radicals (one per DIT) to form the new O-C bond. The mechanism has been calculated for each system with res 374 = D, E, S, K and considering DIT375 acting as acceptor and donor. T4 is obtained via ΔG2, referring to the labeling adopted in the manuscript (see Figure 3). Interestingly, only when the starting T-stacked system is in S=0 BS solution, a low-energy intermediate has been found (here referred to as INT2), in which a bond between the oxygen of the acceptor and the γ-carbon of the donor is formed, but the α-carbon of the latter has not been deprotonated yet.


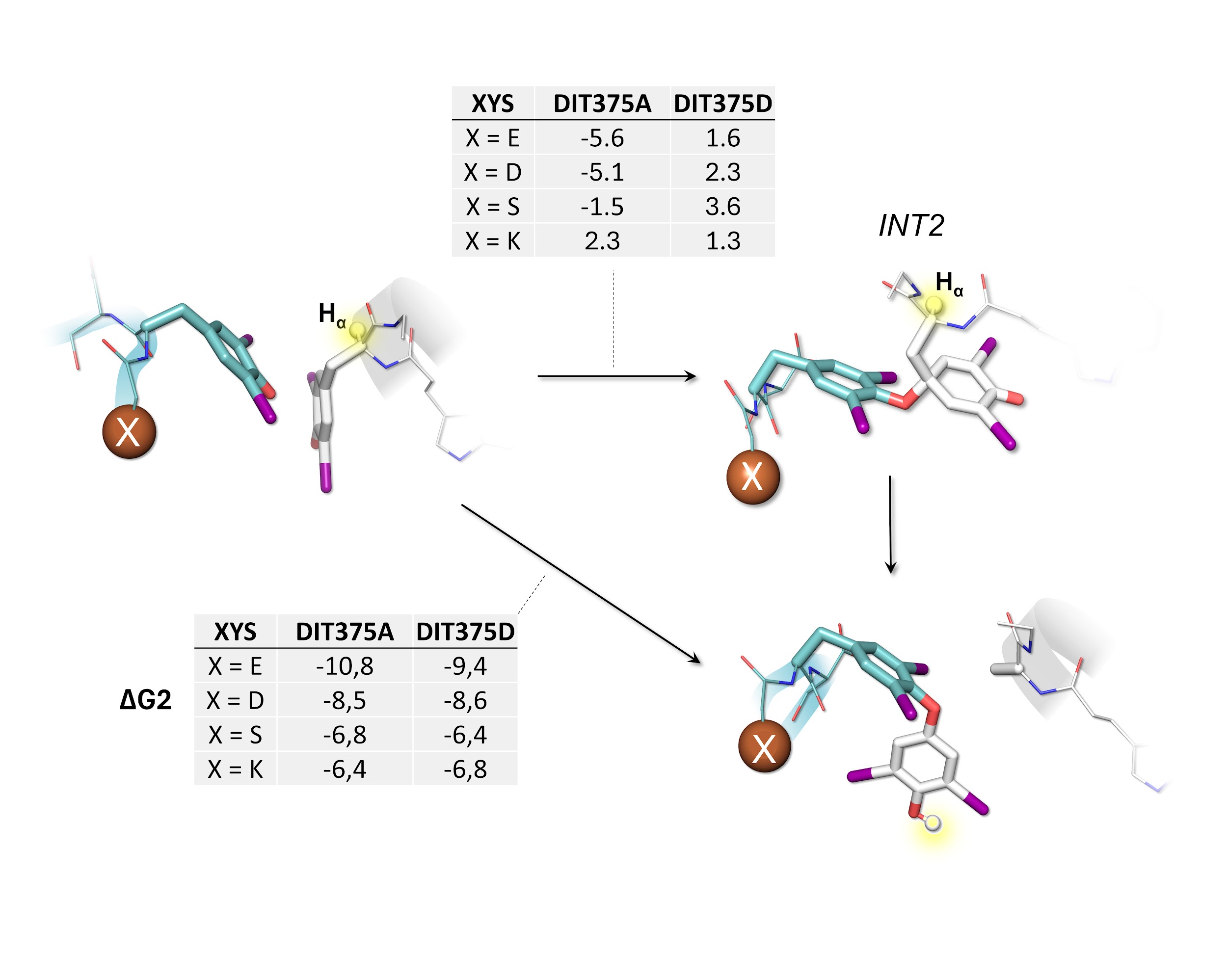


**Figure S11: Natural population analysis relative to tyrosine coupling.**

|  |  | **Charges** | | | |  | **Spin densities** | | | |
| --- | --- | --- | --- | --- | --- | --- | --- | --- | --- | --- |
|  |  | **A** | **B** | **C** | **D** |  | **A** | **B** | **C** | **D** |
| **C** | **1** | 0.07 | 0.07 | 0.05 | 0.02 |  | 0.24 | -0.20 | 0.24 | 0.00 |
|  | **2** | -0.26 | -0.24 | -0.27 | -0.25 |  | -0.04 | 0.02 | -0.05 | 0.00 |
|  | **3** | -0.17 | -0.18 | -0.16 | -0.17 |  | 0.16 | -0.11 | 0.17 | 0.00 |
|  | **4** | 0.28 | 0.28 | 0.30 | 0.15 |  | 0.03 | -0.12 | 0.03 | 0.00 |
|  | **5** | -0.18 | -0.16 | -0.15 | -0.15 |  | 0.14 | -0.14 | 0.20 | 0.00 |
|  | **6** | -0.25 | -0.25 | -0.27 | -0.24 |  | -0.04 | 0.03 | -0.05 | 0.00 |
| **O** | **1** | -0.55 | -0.54 | -0.56 | -0.47 |  | 0.25 | -0.22 | 0.23 | 0.00 |
| **I** | **1** | 0.21 | 0.21 | 0.19 | 0.18 |  | 0.04 | -0.05 | 0.00 | 0.00 |
|  | **2** | 0.21 | 0.22 | 0.15 | 0.19 |  | 0.04 | -0.03 | 0.00 | 0.00 |
| **C’** | **1’** | 0.08 | 0.07 | -0.02 | 0.16 |  | -0.22 | 0.25 | 0.00 | 0.00 |
|  | **2’** | -0.26 | -0.24 | -0.24 | -0.17 |  | -0.03 | -0.05 | 0.00 | 0.02 |
|  | **3’** | -0.17 | -0.17 | -0.19 | -0.23 |  | -0.13 | 0.17 | 0.00 | -0.01 |
|  | **4’** | 0.29 | 0.29 | 0.19 | 0.39 |  | -0.12 | 0.03 | 0.00 | 0.00 |
|  | **5’** | -0.17 | -0.17 | -0.20 | -0.23 |  | -0.15 | 0.16 | 0.00 | -0.01 |
|  | **6’** | -0.26 | -0.27 | -0.24 | -0.17 |  | 0.03 | -0.04 | 0.00 | -0.01 |
| **O’** | **1’** | -0.54 | -0.54 | -0.64 | -0.51 |  | -0.22 | 0.26 | 0.00 | 0.01 |
| **I’** | **1’** | 0.22 | 0.21 | 0.18 | 0.18 |  | -0.05 | 0.04 | 0.00 | 0.00 |
|  | **2’** | 0.23 | 0.19 | 0.20 | 0.18 |  | -0.05 | 0.05 | 0.00 | 0.00 |
| **H** |  |  |  | 0.39 |  |  |  |  | 0.00 |  |

Natural population analysis (NPA) for selected species: doubly oxidized S=0 system with DITs in parallel (A) or perpendicular (B) stacking, singly oxidized system with DITs in perpendicular stacking (C) and the low-energy intermediate (D, see Supplementary Figure S10, INT2).

**
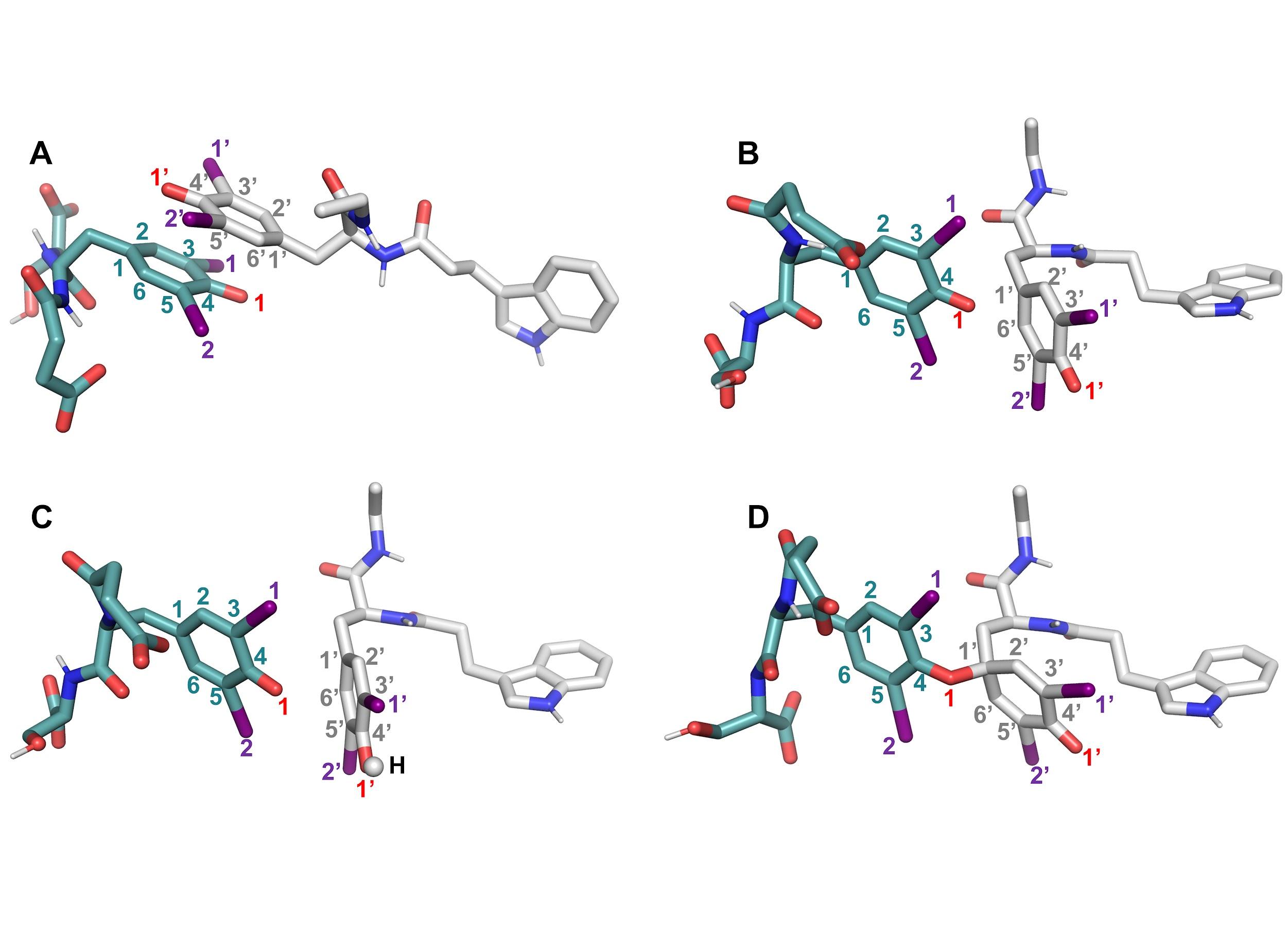
**

**Figure S12: pKa prediction of 2-iodophenol and 2,6-iodophenol.** The calibration linear curve was built considering eight halogen-substituted phenols with known pKA and calculating ΔpKA values (both experimental and theoretical) taking tyrosine as a reference. At pH 7 DIT may be ≈75% ionized, with deprotonation of hydroxyl likely mediated by proximal acidic residues. For instance, the calculated energy of deprotonation of DIT375 by an adjacent carboxylic group (e.g. E) is around 4 kcal/mol, indicating a very facile process at room temperature.


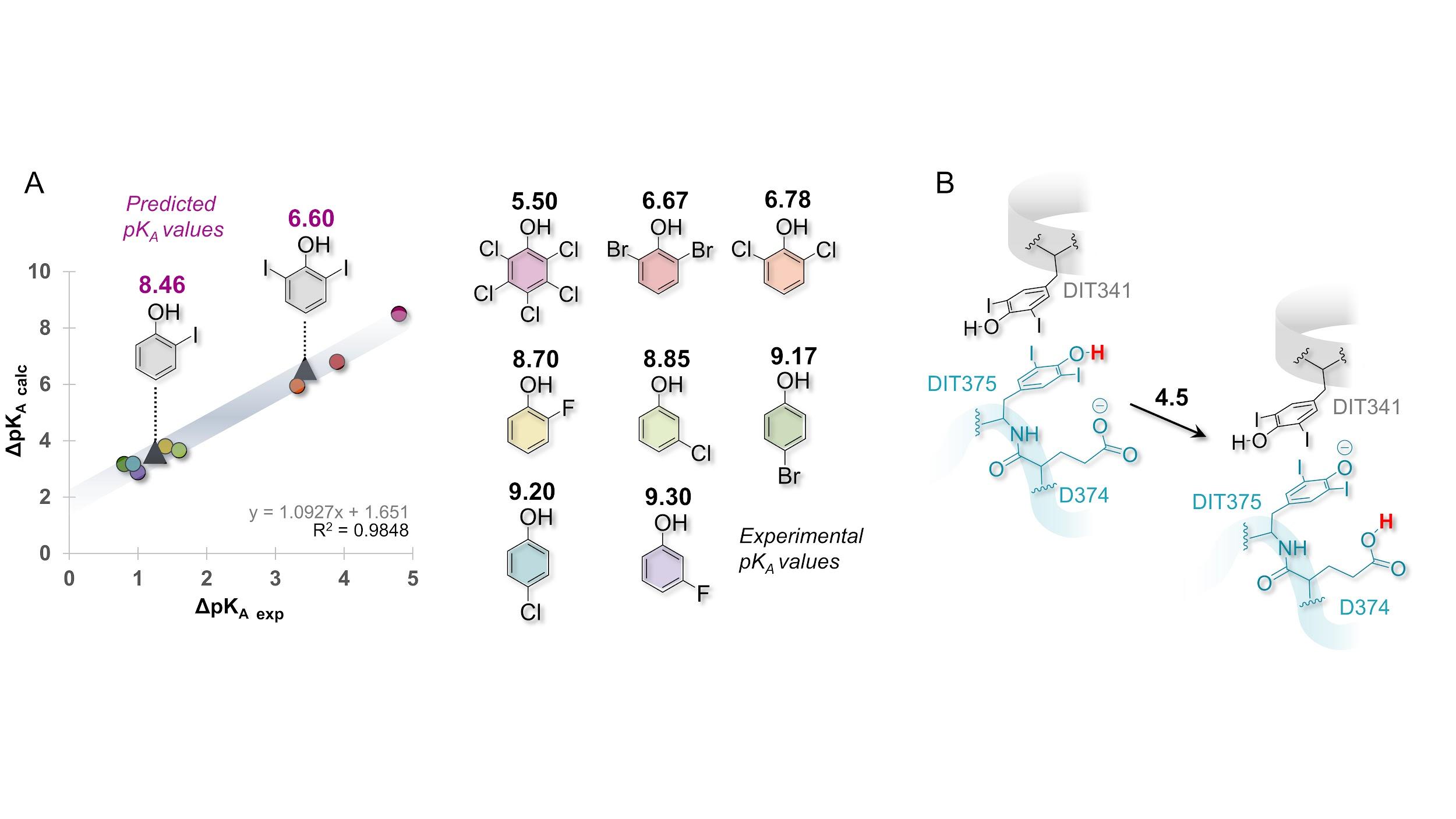


**Figure S13: Calculated energies for radical reaction steps.** Calculated energies for selected steps at the B3LYP/def2-TZVP/COSMO/D4 level of theory (red values). As a comparison, also BP86 results are reported (in parentheses). B3LYP results confirm the same thermodynamic landscape provided by BP86 calculations. S-T (singlet (S=0) - triplet (S=1)) gap has been calculated as the difference between the energy of S=1 state and of S=0 one, so positive values indicate that the singlet state is preferred.


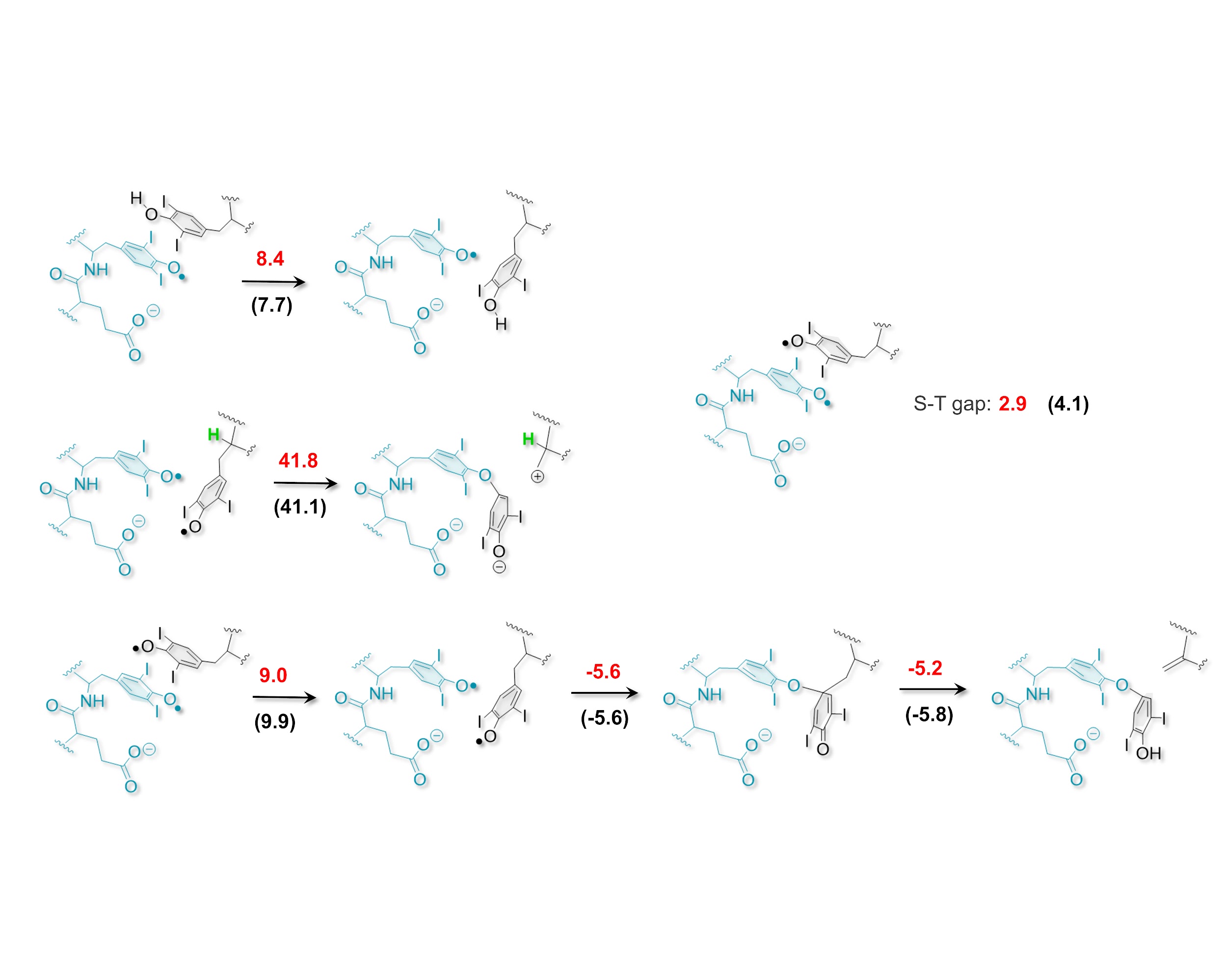


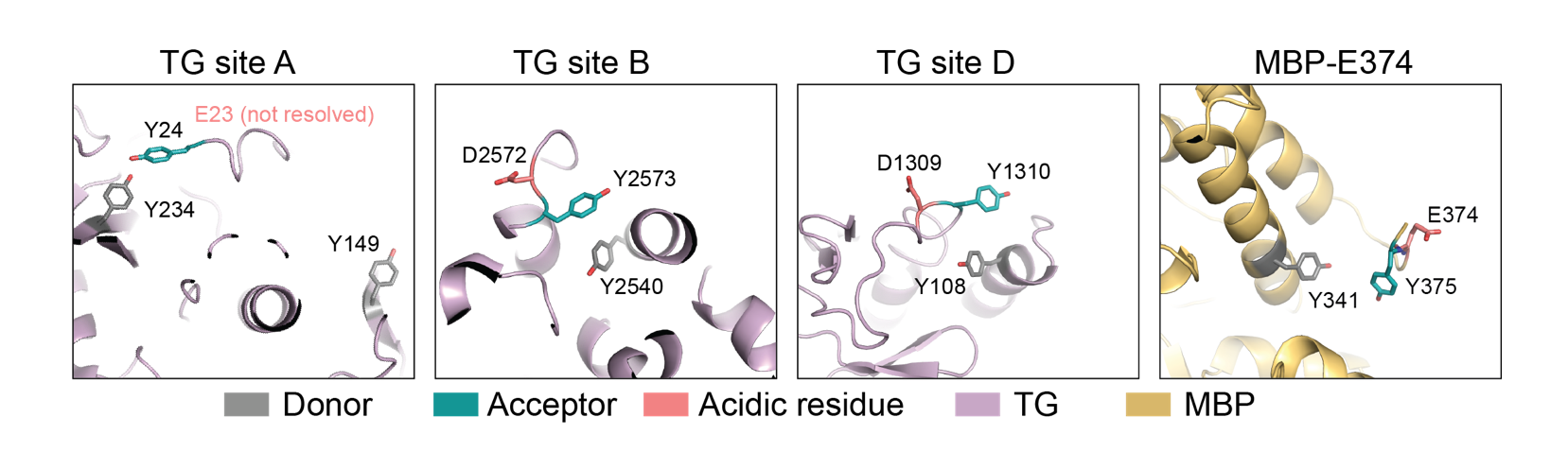
**Figure S14: Comparison between TG and MBP hormonogenic sites.** Close-up views of TG (violet) hormonogenic sites A, B and D and the engineered MBP-E374 (gold) hormonogenic variant containing a single site (acceptor in teal, donor in gray, acidic residue salmon). The TG coordinates refer to PDBID 6SCJ, while MBP coordinates correspond to frame 207 of the Molecular Dynamics (MD) simulation.
